# Engineered Allosteric Biosensor for In Situ DNA Glycosylase Detection in Neuroblastoma Risk Stratification and Drug Resistance Monitoring

**DOI:** 10.1002/advs.77924

**Published:** 2026-09-27

**Authors:** Yaqing Lu, Yingyu Zhang, Xianwei Zhang, Mengxin Zhang, Fei Zhang, Jushan Sun, Yahui Yang, Kangbo Liu, Peng Wang, Shuying Luo, Wancun Zhang

**Affiliations:** ^1^ Health Commission of Henan Province Key Laboratory for Precision Diagnosis and Treatment of Pediatric Tumor Children's Hospital Affiliated to Zhengzhou University Zhengzhou China; ^2^ Henan Key Laboratory of Rare Diseases Endocrinology and Metabolism Center The First Affiliated Hospital, and College of Clinical Medicine of Henan University of Science and Technology Luoyang China; ^3^ State Key Laboratory of Light Superalloys Henan University of Science and Technology Luoyang China; ^4^ College of Public Health Zhengzhou University Zhengzhou P. R. China; ^5^ NMPA Key Laboratory for Quality Evaluation of Medical Protective and Implant Devices Henan Institute for Drug and Medical Device Inspection (Henan Vaccine Issuance Center) Zhengzhou China; ^6^ Department of Biomedical Engineering School of Engineering China Pharmaceutical University Nanjing China

**Keywords:** biosensor, cancer research, dna glycosylase, drug resistance, in vivo, lactate dehydrogenase, machine learning, neuroblastoma, risk stratification

## Abstract

Accurate and in situ detection of uracil‐DNA glycosylase (UDG) is crucial for clinical diagnosis and prognosis assessment. However, conventional methods show low accuracy, limited sensitivity, and off‐target signal leakage due to flawed signal input/output and amplification mechanisms. Therefore, an engineered allosteric biosensor (UUU‐DZ‐tFNA) was developed, in which UDG specifically recognizes and activates a DNAzyme to achieve signal cycle amplification in a sequentially activatable mode. Experimental results demonstrated that UUU‐DZ‐tFNA enables rapid, highly sensitive (LOD = 0.025 mU/mL), and specific detection of UDG. In addition, UUU‐DZ‐tFNA enables in situ molecular imaging of UDG at both the neuroblastoma (NB) cellular and animal levels, as well as precise in situ detection of UDG in plasma exosomes from NB patients. In particular, the non‐invasive risk stratification model for NB developed using machine learning based on exosomal UDG and clinical multidimensional indicators, including *MYCN* amplification status, International NB Risk Factors (IDRFs), neuron‐specific enolase (NSE), and lactate dehydrogenase (LDH), showed excellent discriminatory ability, with 85.7% sensitivity, 100.0% specificity, and 92.8% accuracy. UUU‐DZ‐tFNA also enables image‐guided surgical excision in vivo and effective monitoring of drug resistance in NB. In summary, UUU‐DZ‐tFNA enables in situ detection of UDG, facilitating risk stratification and drug resistance monitoring of NB.

## Graphical abstract

An engineered allosteric biosensor (UUU‐DZ‐tFNA) was developed, in which UDG specifically recognizes and activates a DNAzyme to achieve signal cycle amplification in a sequentially activatable mode. UUU‐DZ‐tFNA achieves in situ UDG detection across multiple biological levels and facilitates risk stratification and drug resistance monitoring of NB.

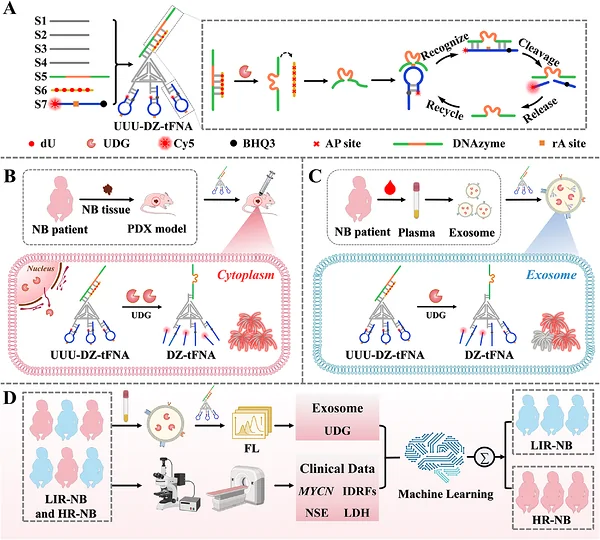

## 1 Introduction

Neuroblastoma (NB) is the most common extracranial solid malignancy in children, representing 6% – 10% of all pediatric cancers but accounting for 15% of childhood cancer‐related deaths [1, 2]. NB exhibits extreme heterogeneity: a subset of tumors may undergo spontaneous regression without intervention, whereas the majority of cases present with widespread systemic metastasis at diagnosis and progress rapidly, resulting in poor patient outcomes [3, 4]. The Children's Oncology Group (COG) stratifies NB into low‐, intermediate‐, and high‐risk subgroups based on multiple clinicopathological and molecular parameters, including *MYCN* copy number, DNA ploidy, International NB Staging System (INSS) stage, and International NB Pathology Classification (INPC) [5]. Clinically, histopathological biopsy of primary or metastatic tumor lesions remains crucial for NB risk stratification. However, most children with high‐risk NB (HR‐NB) are diagnosed at an advanced stage with a large tumor burden, and direct biopsy carries substantial risks, including anesthesia‐related complications, massive intraoperative and postoperative hemorrhage, tumor rupture, and intra‐abdominal infection [6]. Furthermore, *MYCN* copy number, a key NB prognostic marker, requires direct tumor tissue analysis, highlighting the limits of invasive tissue‐dependent stratification [5, 7]. Clinically, the 5‐year survival rate for low‐ and intermediate‐risk NB (LIR‐NB) surpasses 96%, while that for HR‐NB stands at only 48%, highlighting the critical need to improve survival outcomes in this high‐risk population [5, 8]. Clinical practice has proved that timely and accurate risk stratification followed by effective chemotherapy is one of the most effective strategies to improve prognosis for children with HR‐NB [3]. Therefore, establishing a non‐invasive risk stratification strategy for NB that obviates the need for tumor tissue is vital for improving survival rates in children with NB.

Although immunotherapy and targeted therapy have gained significant attention, chemotherapy remains the primary clinical approach for controlling tumor growth, with neoadjuvant or adjuvant chemotherapy serving as a key component of HR‐NB treatment [9, 10]. Most HR‐NB patients respond well to initial chemotherapy. However, more than 60% eventually develop acquired multidrug resistance (MDR), leading to tumor recurrence and/or metastasis and ultimately contributing to pediatric mortality [11]. Thus, acquired MDR during chemotherapy is one of the primary causes of the high mortality rate in HR‐NB. Clinically, drug resistance in NB is comprehensively evaluated based on histopathology (requiring invasive puncture or surgical tissue), blood biomarkers, and medical imaging [12, 13, 14]. However, using tumor recurrence as the criterion for assessing drug resistance is inherently invasive and delayed, resulting in poor diagnostic timeliness. By the time drug resistance is detected, most children have already developed systemic metastasis, missing the optimal therapeutic window [9, 15]. Therefore, existing approaches for early detection of NB drug resistance cannot meet clinical demands. There is an urgent need to develop novel strategies for monitoring NB drug resistance.

The base excision repair (BER) pathway preserves genomic stability by correcting damaged or chemically modified nucleobases, such as those induced by oxidative stress, alkylating agents, or ionizing radiation [16]. BER is initiated by specialized DNA glycosylases, such as UDG, which recognize and excise damaged or inappropriate bases to generate AP sites [17, 18]. These AP sites are subsequently incised by apurinic/apyrimidinic endonuclease 1 (APE1), producing single‐strand breaks that serve as substrates for downstream BER enzymes [19, 20]. As an essential initiating enzyme of the BER pathway, UDG plays a critical role in maintaining genomic stability. Recent studies have demonstrated aberrant upregulation of UDG in a spectrum of malignant tumors such as lung cancer and colorectal cancer, which exhibits a strong correlation with tumor chemoresistance [21, 22, 23]. Mechanistically, UDG facilitates enhanced DNA repair capacity to counteract DNA lesions induced by chemotherapeutic agents, ultimately mediating the acquisition of drug‐resistant phenotypes. Furthermore, emerging clinical and preclinical data validate UDG as a prognostic biomarker for tumor risk stratification. In non‐small cell lung cancer, elevated UDG independently predicts reduced progression‐free and overall survival, and concurrent upregulation of UDG and BRCA1 identifies the highest‐risk subgroup treated with pemetrexed [24]. For colorectal cancer, UDG enzymatic activity is markedly elevated in metastatic advanced lesions relative to early localized tumors. Excessively activated UDG‐dependent BER eliminates uracil lesions induced by 5‐FU, conferring chemoresistance exclusively in high‐risk colorectal tumors [25]. Accumulating evidence indicates that aberrant BER activity contributes to genomic instability, tumorigenesis, and therapeutic resistance [26, 27]. Our previous research demonstrated that APE1 is a reliable biomarker for predicting NB risk and drug resistance [28, 29]. Given that UDG acts upstream of APE1 in the BER pathway, we focused on UDG and examined its correlation with NB risk stratification and drug resistance.

To systematically evaluate the role of UDG in NB risk stratification and drug resistance monitoring, a highly sensitive and specific in situ detection method for UDG is required. Over the past few years, UDG detection technologies have evolved into a diversified system, ranging from traditional to novel sensing strategies and from in vitro to live‐cell analysis [30, 31, 32, 33]. In recent years, nucleic acid isothermal amplification, CRISPR‐Cas based targeted recognition, and enzyme cascade amplification have enhanced the sensitivity of UDG detection, enabling quantitative analysis at the cellular level and supporting research on enzyme function and inhibitor screening [34, 35, 36, 37, 38]. Although various UDG detection methods have been reported, significant limitations remain for practical applications. First, traditional methods, including radiolabeled gel electrophoresis and mass spectrometry, suffer from labor‐intensive protocols, poor sensitivity, and safety hazards. These methods are limited to analyzing UDG in cell lysates, failing to capture real‐time molecular information. Consequently, lysate‐based measurements may misrepresent the true status of UDG in live cells, resulting in inaccurate assessment [39, 40]. Second, for most in situ UDG detection approaches, signal output relies on the cleavage of the resulting AP sites by APE1. Consequently, the signal intensity depends not only on UDG levels but also on APE1 levels in cells, which may confound the quantitative results of these methods [31, 38]. Thirdly, the few studies that achieve independent UDG detection exhibit relatively low sensitivity [41, 42, 43, 44, 45]. Therefore, a method for highly sensitive in situ UDG detection is urgently needed to accurately capture UDG‐related molecular information for disease diagnosis and mechanistic studies.

**SCHEME 1:**
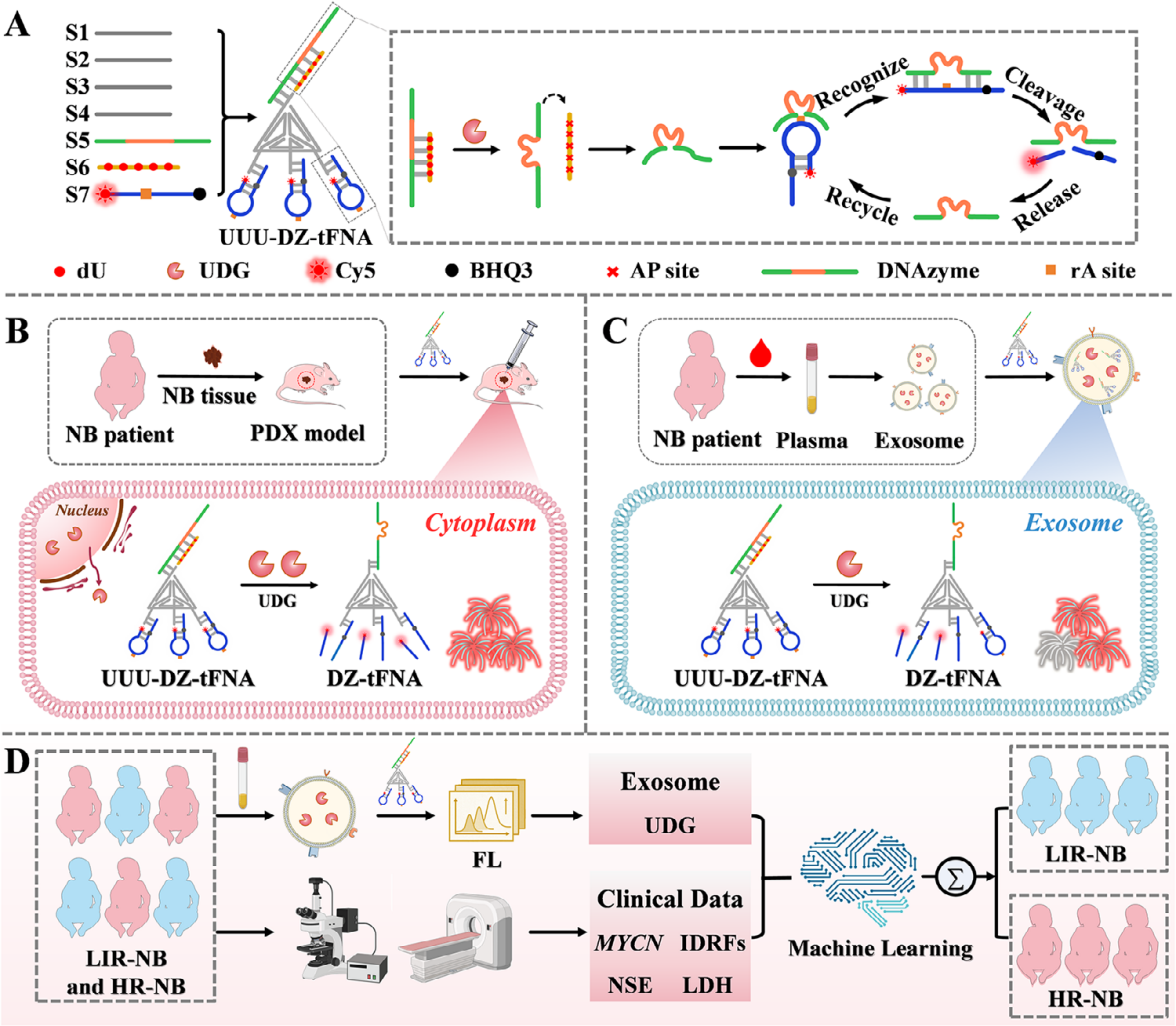
(A) Schematic illustration of the synthesis and reaction principle of UUU‐DZ‐tFNA for UDG detection. (B) Schematic representation of in vivo imaging of UDG using UUU‐DZ‐tFNA. (C) Schematic depiction of exosomal UDG detection by UUU‐DZ‐tFNA. (D) Schematic overview of the construction of a non‐invasive risk stratification model for NB using machine learning based on exosomal UDG and clinical multidimensional indicators. The schematic diagram was designed by the authors. Partial icons used in this figure are adapted from BioRender asset library (https://biorender.com).

Therefore, this study developed an engineered allosteric biosensor (UUU‐DZ‐tFNA) for high specificity and high sensitivity in situ imaging of UDG. UUU‐DZ‐tFNA, synthesized via self‐assembly, comprises a tetrahedral framework DNA (tFNA) formed from the hybridization of strands S1 – S4, a double‐stranded DNA formed from the hybridization of strands S5 and S6, and a thermally stable hairpin (HP2, S7). Owing to the excellent cell membrane and tissue permeability of UUU‐DZ‐tFNA, it can efficiently penetrate the cell membranes of both tumor and normal cells via small pocket‐mediated and lysosome‐mediated endocytosis pathways [28, 46, 47]. The mechanism of UUU‐DZ‐tFNA response to UDG is described in Scheme 1A. In the presence of UDG, the dU base in the lock chain (S6) is specifically removed, reducing base pairing stability and leading to double‐helix unwinding and conformational changes in the DNAzyme. Consequently, the fully exposed active region of the DNAzyme recovers its catalytic activity, specifically recognizing and cleaving the rA site on HP2 (S7), thereby generating a fluorescence signal. Notably, the reactivated DNAzyme chain can repeatedly bind to other tetrahedral vertices, enabling cyclic amplification of the fluorescence signal (Scheme 1A). Owing to caveolin‐mediated endocytosis [48, 49] and passive transmembrane diffusion [50, 51], UUU‐DZ‐tFNA can not only penetrate cell membranes but also enter exosomes, thereby enabling in situ detection of UDG in vivo and in exosomes (Scheme 1B,C). To achieve non‐invasive risk stratification of NB, this study developed a machine learning‐based non‐invasive risk stratification model integrating exosomal UDG and clinical multidimensional indicators, including *MYCN* amplification status, International NB Risk Factors (IDRFs), neuron‐specific enolase (NSE), and lactate dehydrogenase (LDH) (Scheme 1D). The key innovations of this study are summarized below. (1) Unique signal input/output and amplification mechanisms. UUU‐DZ‐tFNA is specifically recognized by UDG, activating a DNAzyme for sequential signal cycle amplification. (2) Excellent detection performance. UUU‐DZ‐tFNA achieves highly sensitive and specific in situ UDG detection at the NB cellular, animal, and exosome levels. (3) Broad prospects for clinical application. UUU‐DZ‐tFNA holds promise for non‐invasive NB risk stratification and drug resistance monitoring. Therefore, UUU‐DZ‐tFNA serves as a valuable tool for simple, sensitive, and specific in situ detection of UDG across multiple scales through a dual‐enzyme‐mediated, sequentially activated mechanism, holding significant promise for noninvasive risk stratification and drug resistance monitoring in NB.

## 2 Results and Discussion

### 2.1 UDG Expression in NB Cells, Tissues, and Plasma

**FIGURE 1:**
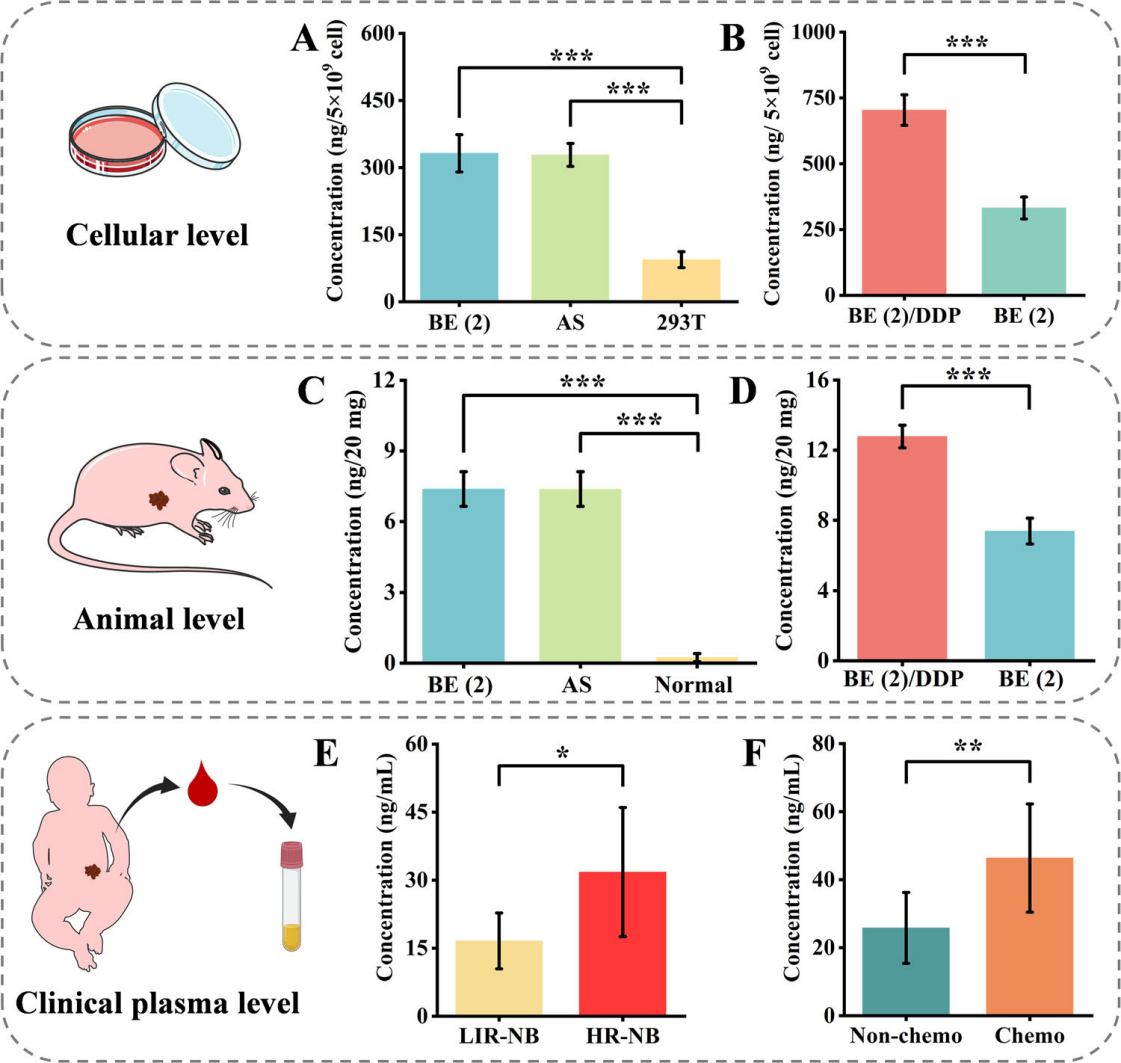
UDG expression in NB cells, animal models, and clinical samples was quantified using a UDG ELISA kit. (A) Cytoplasmic UDG levels in NB cells versus normal cells. (B) Cytoplasmic UDG levels in SK‐N‐BE(2)/DDP and SK‐N‐BE(2) cells. (C) Cytoplasmic UDG levels in tumor tissues from NB CDX models and normal tissues. (D) Cytoplasmic UDG levels in tumor tissues from SK‐N‐BE(2)/DDP and SK‐N‐BE(2) CDX models. (E) Plasma UDG levels in LIR‐NB and HR‐NB patients. (F) Plasma UDG levels of NB patients before (Non‐chemo) and after (Chemo) multiple cycles of chemotherapy. Data are presented as mean ± SD (*n* = 3 for A—D; *n* = 13 for E; *n* = 10 for F). *P < 0.05; **P < 0.01; ***P < 0.001. Abbreviations: BE (2), SK‐N‐BE (2); AS, SK‐N‐AS; BE (2)/DDP, SK‐N‐BE (2)/DDP.

To evaluate the utility of UDG for non‐invasive risk stratification and drug resistance monitoring of NB, we quantitatively measured UDG expression in NB cells, tissues, and plasma using a classic enzyme‐linked immunosorbent assay (ELISA) kit. Experimental results showed that cytoplasmic UDG expression levels in NB cell lines, SK‐N‐BE (2) and SK‐N‐AS, were significantly higher than those in normal 293T cells (Figure 1A). Furthermore, cytoplasmic UDG expression in drug‐resistant SK‐N‐BE (2) cells was significantly higher than that in parental SK‐N‐BE (2) cells (Figure 1B). In cell‐derived xenograft (CDX) models, cytoplasmic UDG expression in tumor tissues from SK‐N‐BE (2) and SK‐N‐AS tumor‐bearing mice was significantly higher than that in normal tissues (Figure 1C). Additionally, cytoplasmic UDG expression in tumor tissues from SK‐N‐BE (2)/DDP tumor‐bearing mice was significantly higher than that from SK‐N‐BE (2) tumor‐bearing mice (Figure 1D). To further validate the association between UDG and NB risk stratification, we quantified plasma UDG concentrations in patients stratified as HR‐NB or LIR‐NB according to established clinical criteria (Figure 1E). Plasma UDG levels were significantly elevated in HR‐NB relative to LIR‐NB. Moreover, patients displayed markedly higher plasma UDG after multiple cycles of chemotherapy compared with their pretreatment baseline values (Figure 1F). Therefore, UDG is a potential biomarker for NB risk stratification and drug resistance monitoring.

### 2.2 Synthesis and Characterization of UUU‐DZ‐tFNA

**FIGURE 2:**
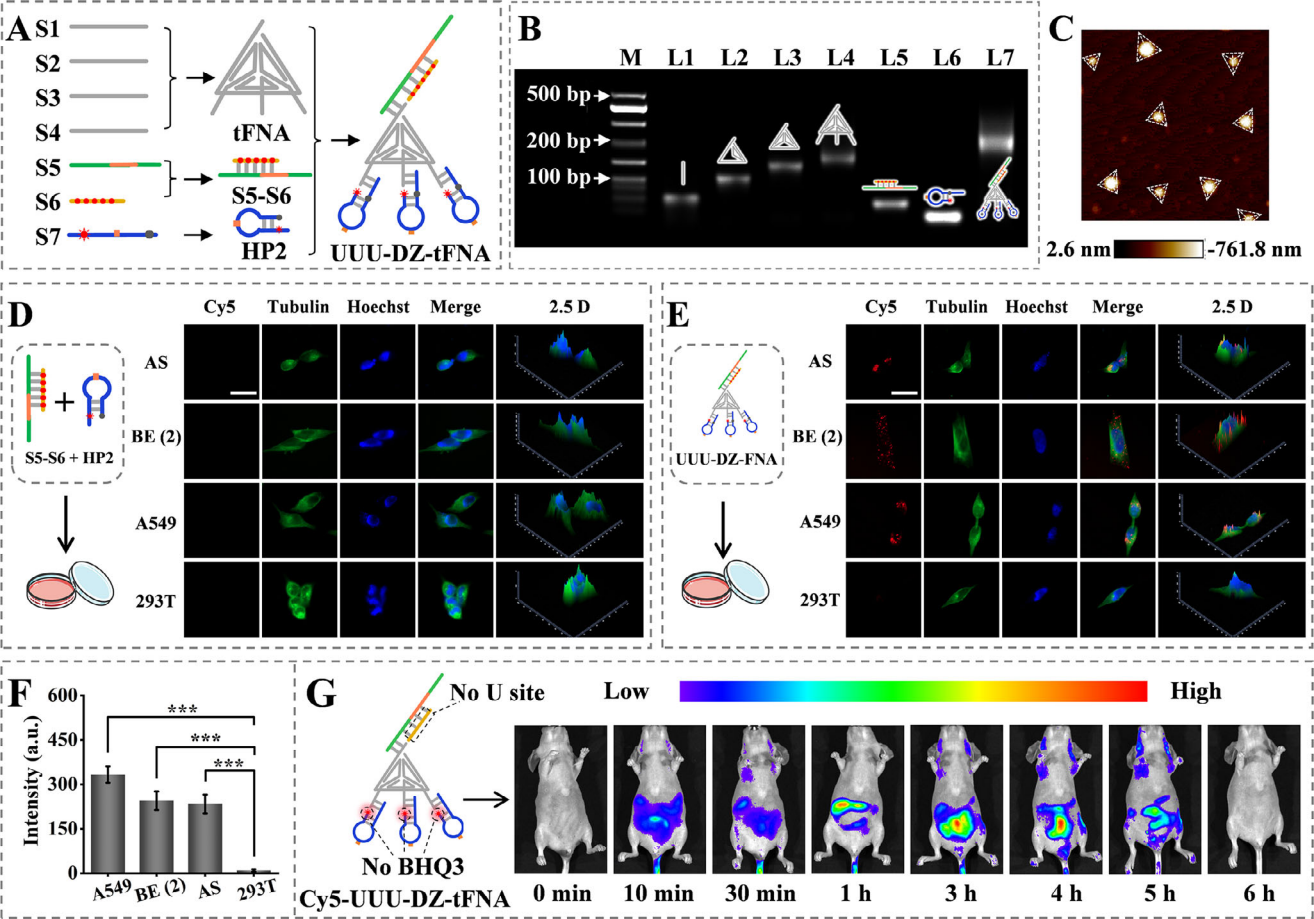
Synthesis and characterization of UUU‐DZ‐tFNA. (A) Schematic illustration of the self‐assembly process for UUU‐DZ‐tFNA. (B) The successful formation of UUU‐DZ‐tFNA was confirmed by 1.5% AGE. M, DNA marker; L1, S1; L2, S1 + S2; L3, S1 + S2 + S3; L4, tFNA; L5, S5‐S6; L6, HP2 (S7); L7, UUU‐DZ‐tFNA. (C) AFM revealed the morphology and dimensions of UUU‐DZ‐tFNA. Scale bar: 50 nm. (D) Fluorescence microscopy images of SK‐N‐AS, SK‐N‐BE (2), A549, and 293T cells treated with S5‐S6 and HP2. Scale bar: 40 µm. (E) Fluorescence microscopy images of SK‐N‐AS, SK‐N‐BE (2), A549, and 293T cells incubated with UUU‐DZ‐tFNA; (F) corresponding quantitative fluorescence signal histogram. Scale bar: 40 µm. (G) In vivo fluorescence imaging of nude mice was performed at multiple time points after intravenous administration of UUU‐DZ‐tFNA. Data are presented as mean ± SD (*n* = 3). ***P < 0.001.

To systematically evaluate the role of UDG in NB risk stratification and drug resistance monitoring, a robust, highly sensitive, and specific method for in situ UDG detection is essential. Therefore, the synthesis and characterization of UUU‐DZ‐tFNA were systematically studied, as its successful synthesis represents a critical prerequisite for efficient cellular internalization and subsequent in vivo imaging applications [52, 53, 54]. UUU‐DZ‐tFNA is synthesized based on the principle of base complementary pairing and consists of S1 – S7 (Table S1). As illustrated in Figure 2A, equimolar amounts of the four single‐stranded DNAs (S1 – S4) self‐assembled into tFNA following the principle of base complementary pairing. Subsequently, the S5‐S6 duplex was generated by annealing S5 and S6 at a 1:1 molar ratio. Finally, UUU‐DZ‐tFNA was constructed by mixing tFNAs, the S5‐S6 duplex, and S7 at a 1:1:3 molar ratio, followed by incubation at 37°C for 30 min to drive self‐assembly. The synthesis and characterization of UUU‐DZ‐tFNA were then performed using a variety of analytical techniques. Agarose gel electrophoresis (AGE) confirmed the successful fabrication of UUU‐DZ‐tFNA (Figure 2B), a finding further validated by capillary electrophoresis analysis (Figure S1). Atomic force microscopy (AFM) imaging revealed that UUU‐DZ‐tFNA adopted a uniform triangular morphology with an average particle size of 44.80 ± 1.46 nm, which is greater than our previously synthesized tFNA (Figure 2C). This size increase likely results from S5‐S6 and S7 modifications at the tFNA vertices [55]. Furthermore, the structural integrity and biofunctionality of UUU‐DZ‐tFNA were verified through comparative cellular UDG imaging assays, contrasting its performance with that of S5‐S6 and S7. As presented in Figure 2D, S5‐S6 and S7 did not elicit detectable fluorescent signals in the NB cell lines SK‐N‐AS and SK‐N‐BE (2), the lung cancer cell line A549, or the human embryonic kidney cell line 293T. In contrast, UUU‐DZ‐tFNA generated robust fluorescent signals in tumor cells, with no discernible signal observed in normal cells (Figure 2E,F), further confirming the successful assembly and target‐responsive activity of the probe. Meanwhile, cellular imaging demonstrated significantly elevated UDG activity in both NB and A549 tumor cells relative to normal 293T cells. Consistent with published evidence, UDG upregulation represents a general feature of diverse malignancies rather than a unique biomarker specific to NB. To interrogate the in vivo metabolic biodistribution of UUU‐DZ‐tFNA, a Cy5‐labeled control probe was synthesized (Cy5‐UUU‐DZ‐tFNA), which lacks both the dU modification site and the BHQ3 quencher moiety. Biodistribution profiling demonstrated that Cy5‐UUU‐DZ‐tFNA was primarily cleared via hepatic and renal pathways, manifesting rapid and efficient systemic metabolism (Figure 2G). In addition, AGE analysis confirmed that UUU‐DZ‐tFNA retained complete structural integrity after incubation with 10% fetal bovine serum (FBS) at 37°C for 24 h, with no observable degradation The construct also exhibited uncompromised stability across a physiologically relevant temperature range (4°C–37°C) over 24 h, verifying its robust resistance to enzymatic and thermal degradation (Figure S2). In summary, the principal findings of this study are as follows: (1) UUU‐DZ‐tFNA was successfully synthesized; (2) UUU‐DZ‐tFNA enables target‐specific detection in cells without the need for transfection reagents; (3) UUU‐DZ‐tFNA is rapidly cleared via hepatic and renal pathways.

### 2.3 UUU‐DZ‐tFNA for Responding UDG In Vitro

**FIGURE 3:**
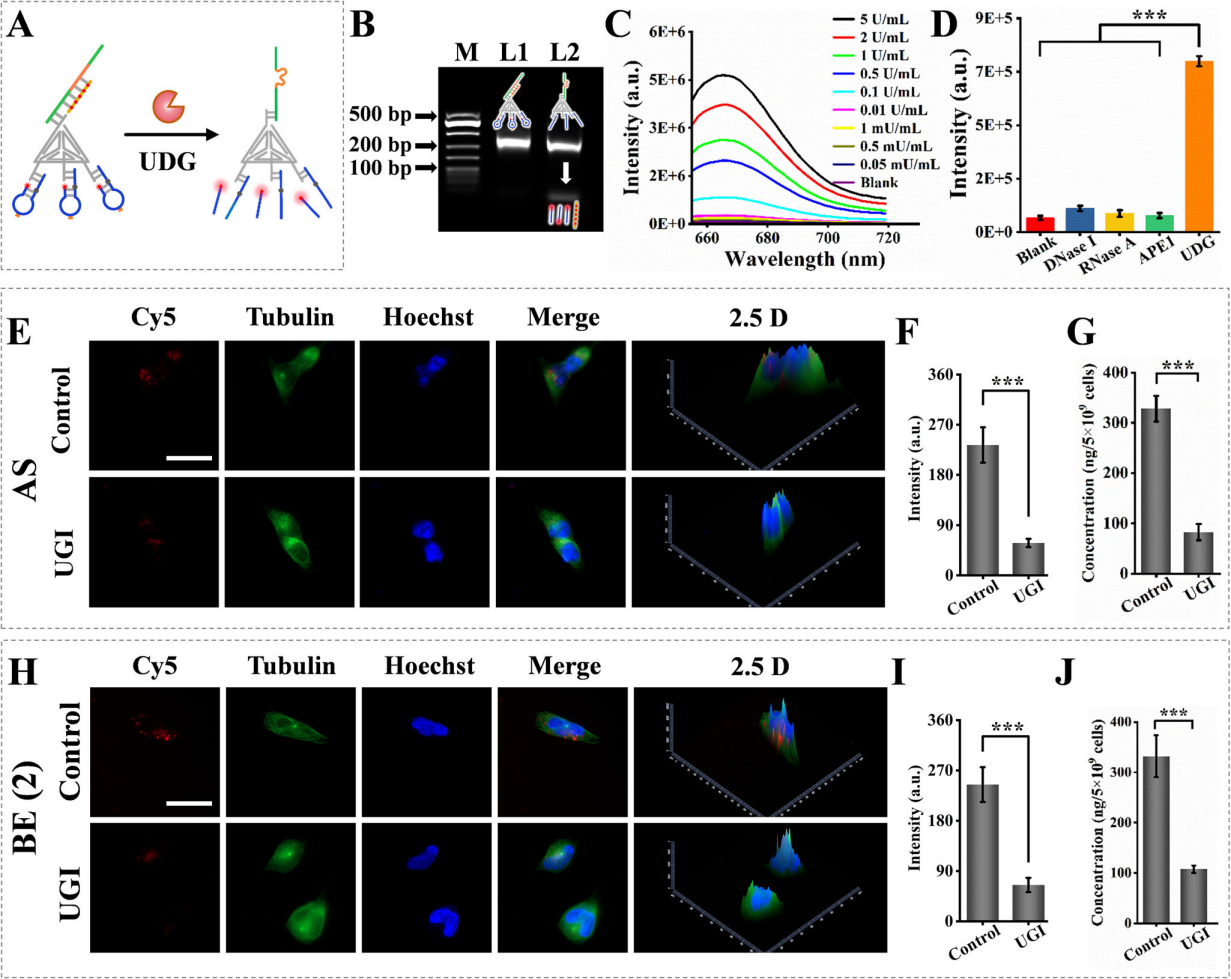
In vitro detection of UDG using UUU‐DZ‐tFNA. (A) Flowchart illustrating the UDG detection procedure based on UUU‐DZ‐tFNA. (B) The response of UUU‐DZ‐tFNA to UDG was analyzed by 1.5% AGE. M, DNA marker; L1, UUU‐DZ‐tFNA; L2, UUU‐DZ‐tFNA + UDG. (C) Fluorescence spectra of UUU‐DZ‐tFNA in response to increasing concentrations of UDG. (D) Specificity of UUU‐DZ‐tFNA for UDG detection. (E) Fluorescence microscopy images of SK‐N‐AS cells in the UGI‐treated group versus the control group, and (F) the corresponding quantitative fluorescence intensity histogram. Scale bar: 40 µm. (G) Cytoplasmic UDG levels in the cells shown in (E) were quantitatively measured using a UDG ELISA kit. (H) Fluorescence microscopy images of SK‐N‐BE (2) cells with or without UGI treatment, and (I) the corresponding quantitative fluorescence intensity histogram. Scale bar: 40 µm. (J) Cytoplasmic UDG levels in the cells shown in (H) measured by a UDG ELISA kit. Data are presented as mean ± SD (*n* = 3). ***P < 0.001.

Given the inherent complexity of biological systems and the low abundance of UDG, there is an urgent need for highly specific and sensitive strategies for in situ UDG detection [37, 45]. Figure 3A illustrates the reaction mechanism of UUU‐DZ‐tFNA in response to UDG. AGE analysis revealed that UUU‐DZ‐tFNA (Figure 3B) underwent a distinct conformational transition upon UDG addition, yielding characteristic cleavage fragments (white arrows in Figure 3B L2). Additional AGE assays confirmed that conformational transformation merely occurred at the S5‐S6 region (L2 site in Figure S3) after UDG treatment, with specific generated fragments indicated by blue arrows at the L6 site in Figure S3. This observation indicates that the signal response of UUU‐DZ‐tFNA to UDG is mediated by UDG‐induced enzymatic cleavage, a finding further validated by capillary electrophoresis results (Figure S4A,B). Kinetic profiling demonstrated that the fluorescence intensity of UUU‐DZ‐tFNA increased progressively in the presence of UDG, reaching a plateau at 3 h. In contrast, negligible changes in fluorescence signal were observed throughout the entire observation period in the absence of UDG (Figure S5A). Furthermore, by adjusting the ratio of S5‐S6 and HP2, it was verified that the high response signal of UUU‐DZ‐tFNA to UDG was attributed to the cyclic amplification mechanism of the DNAzyme (Figure S5B). As presented in Figure 3C, the fluorescence intensity of UUU‐DZ‐tFNA exhibited a robust concentration‐dependent relationship with UDG. A calibration curve for quantitative UDG detection was established: *Y* = 2985127.91 + 2164269.85 1 g C, where *Y* denotes the fluorescence intensity and *C* represents the UDG concentration. The correlation coefficient (R^2^) was 0.99, and the limit of detection (LOD, calculated as 3σ) was 0.025 mU/mL (Figure 3C and Figure S6). This LOD is superior to that of several previously reported UDG detection methods, a superiority attributed to the signal amplification capability of the DNAzyme, which facilitates precise identification of low‐abundance UDG (Table S2) [37, 43, 45, 56, 57, 58]. To confirm the detection specificity of UUU‐DZ‐tFNA, we assessed the potential interference of other nucleases on UDG detection. As shown in Figure 3D, the fluorescence intensity of the UDG group (orange bars) was significantly higher than that of all other control groups, verifying the high specificity of UUU‐DZ‐tFNA for UDG. To further validate the capability of UUU‐DZ‐tFNA for accurate UDG detection in NB cells, we pretreated NB cells with uracil glycosylase inhibitor (UGI), a specific inhibitor of UDG. Prior to fluorescence detection, we first performed CCK‐8 assays to exclude potential cytotoxicity of UGI. No statistically significant differences in cell viability were observed among cells treated with 0, 0.5, 1, and 2 U/mL UGI after 30 min incubation at 37°C (*P* > 0.05) (Figure S7), demonstrating that the selected UGI treatment regimen had negligible cellular toxicity and would not confound subsequent signal readouts. Subsequent fluorescence imaging across two NB cell lines, SK‐N‐AS and SK‐N‐BE(2), revealed a pronounced attenuation of UUU‐DZ‐tFNA fluorescence intensity in UGI‐pretreated groups relative to untreated control cells (Figure 3E,H). To verify the feasibility of UUU‐DZ‐tFNA for UDG detection, UDG was extracted from both UGI‐pretreated and control groups and quantified using a UDG ELISA kit. The results showed that the UDG level in the UGI‐pretreated group was significantly lower than that in the control group, consistent with the fluorescence imaging data (Figure 3F,G,I,J). Collectively, these findings demonstrate that UUU‐DZ‐tFNA enables accurate UDG detection.

### 2.4 UUU‐DZ‐tFNA for the Imaging of UDG in Living Cells

**FIGURE 4:**
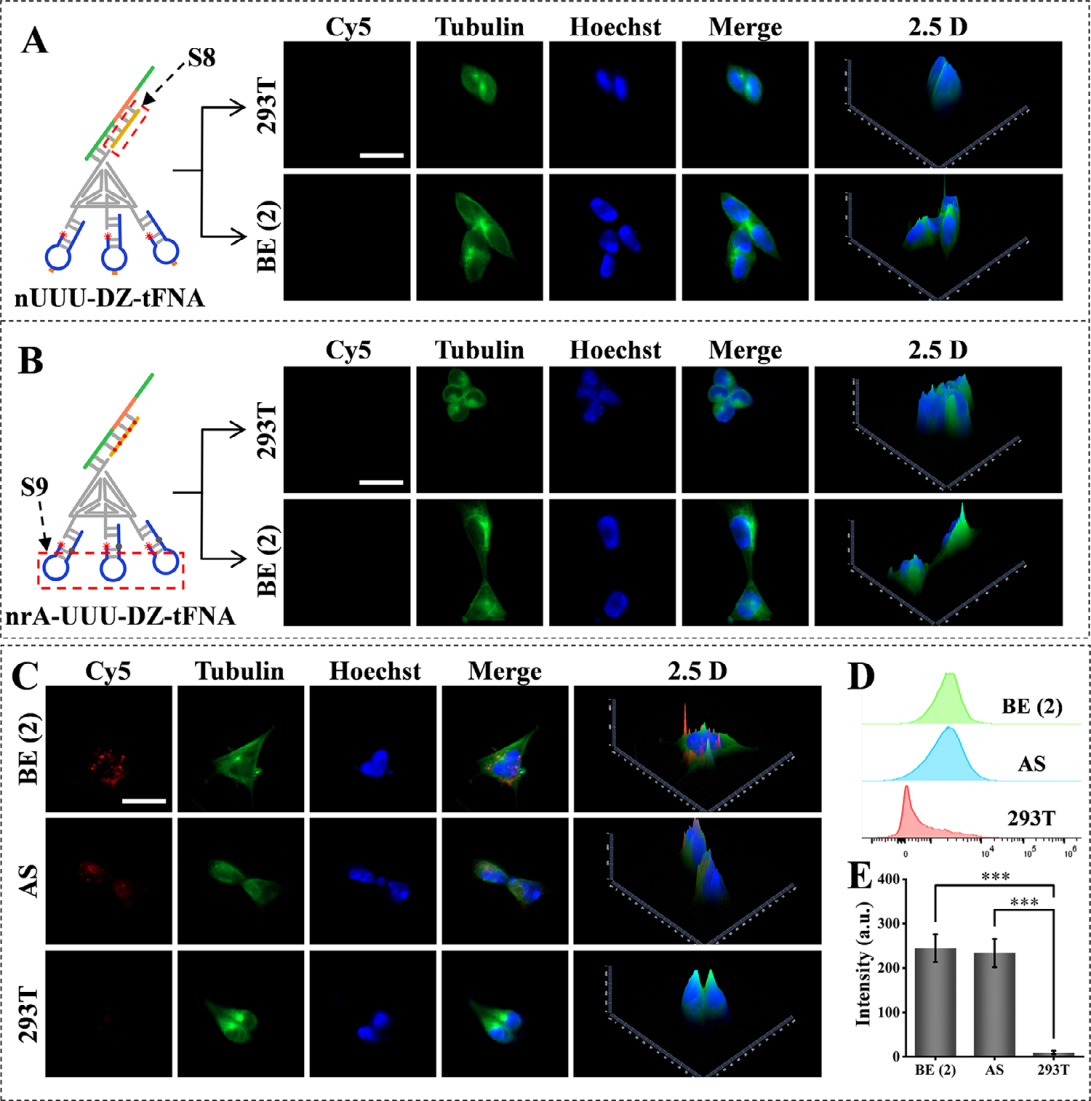
UUU‐DZ‐tFNA for imaging of UDG in living cells. Fluorescence microscopy images of SK‐N‐BE (2) and 293T cells incubated with (A) nUUU‐DZ‐tFNA (S8 in place of S6) and (B) nrA‐UUU‐DZ‐tFNA (rA cleavage site deleted). (C) Fluorescence microscopy images of SK‐N‐AS, SK‐N‐BE (2), and 293T cells treated with UUU‐DZ‐tFNA. Scale bar: 40 µm. (D) Flow cytometry results of UUU‐DZ‐tFNA in SK‐N‐AS, SK‐N‐BE (2), and 293T. (E) Quantitative analysis of Cy5 fluorescence signals in SK‐N‐AS, SK‐N‐BE (2), and 293T cells incubated with UUU‐DZ‐tFNA. Data are presented as mean ± SD (*n* = 3). ***P < 0.001.

Based on the excellent in vitro UDG detection performance of UUU‐DZ‐tFNA, we next systematically investigated its ability for in situ molecular imaging of UDG at the cellular level. Cellular internalization properties are pivotal to enhancing the imaging efficacy of UUU‐DZ‐tFNA at the cellular level [59, 60, 61]. As illustrated in Figure S8, the intracellular fluorescence signal of UUU‐DZ‐tFNA increased progressively with prolonged incubation time, reaching a maximum at 6 h. Thus, a 6‐h incubation period was selected for subsequent cellular imaging experiments. Moreover, the biocompatibility of UUU‐DZ‐tFNA is fundamental to its utility in situ molecular imaging of UDG [60, 62, 63]. CCK‐8 based cell proliferation assays further confirmed that UUU‐DZ‐tFNA exerted no significant inhibitory effect on cell proliferation across a concentration range of 0 – 200 nM, verifying its excellent safety profile for cellular applications (Figure S9). To validate the functional role of the dU sites in UUU‐DZ‐tFNA for mediating interactions with UDG, we designed a control probe (nUUU‐DZ‐tFNA) with modified dU site abundance (Figure 4A). The results showed that nUUU‐DZ‐tFNA, which lacks dU sites, exhibited no detectable signal output, indicating that UDG‐mediated unwinding of the S5‐S6 duplex is essential for triggering the signal amplification cascade in UUU‐DZ‐tFNA. Similarly, to confirm the criticality of the rA site for DNAzyme activation, we constructed another control probe (nrA‐UUU‐DZ‐tFNA) devoid of the rA site (Figure 4B). Consistent with the above findings, nrA‐UUU‐DZ‐tFNA failed to generate a detectable fluorescence signal, demonstrating that the rA site is indispensable for generating fluorescence signals. To evaluate the feasibility of UUU‐DZ‐tFNA for imaging UDG in living cells, we used it to assess UDG expression levels in normal 293T cells and NB tumor cell lines (SK‐N‐BE (2) and SK‐N‐AS). Inverted fluorescence microscopy revealed negligible Cy5‐derived signals (from UUU‐DZ‐tFNA) in 293T cells, whereas robust and distinct signals were observed in both SK‐N‐BE (2) and SK‐N‐AS cells (Figure 4C). Flow cytometry analysis further corroborated these observations (Figure 4D). To further confirm the accuracy of UUU‐DZ‐tFNA for UDG detection in living cells, we assessed UDG expression in 293T, SK‐N‐BE (2), and SK‐N‐AS cells. Quantitative analyses, including fluorescence intensity quantification and ELISA, consistently demonstrated significantly higher UDG expression levels in SK‐N‐BE (2) and SK‐N‐AS cells compared to 293T cells (*P* < 0.001) (Figure 4E and Figure S10). Taken together, these data establish that UUU‐DZ‐tFNA provides robust UDG imaging performance and effectively differentiates tumor cells from their normal counterparts.

### 2.5 UUU‐DZ‐tFNA for In Vivo Imaging of UDG

**FIGURE 5:**
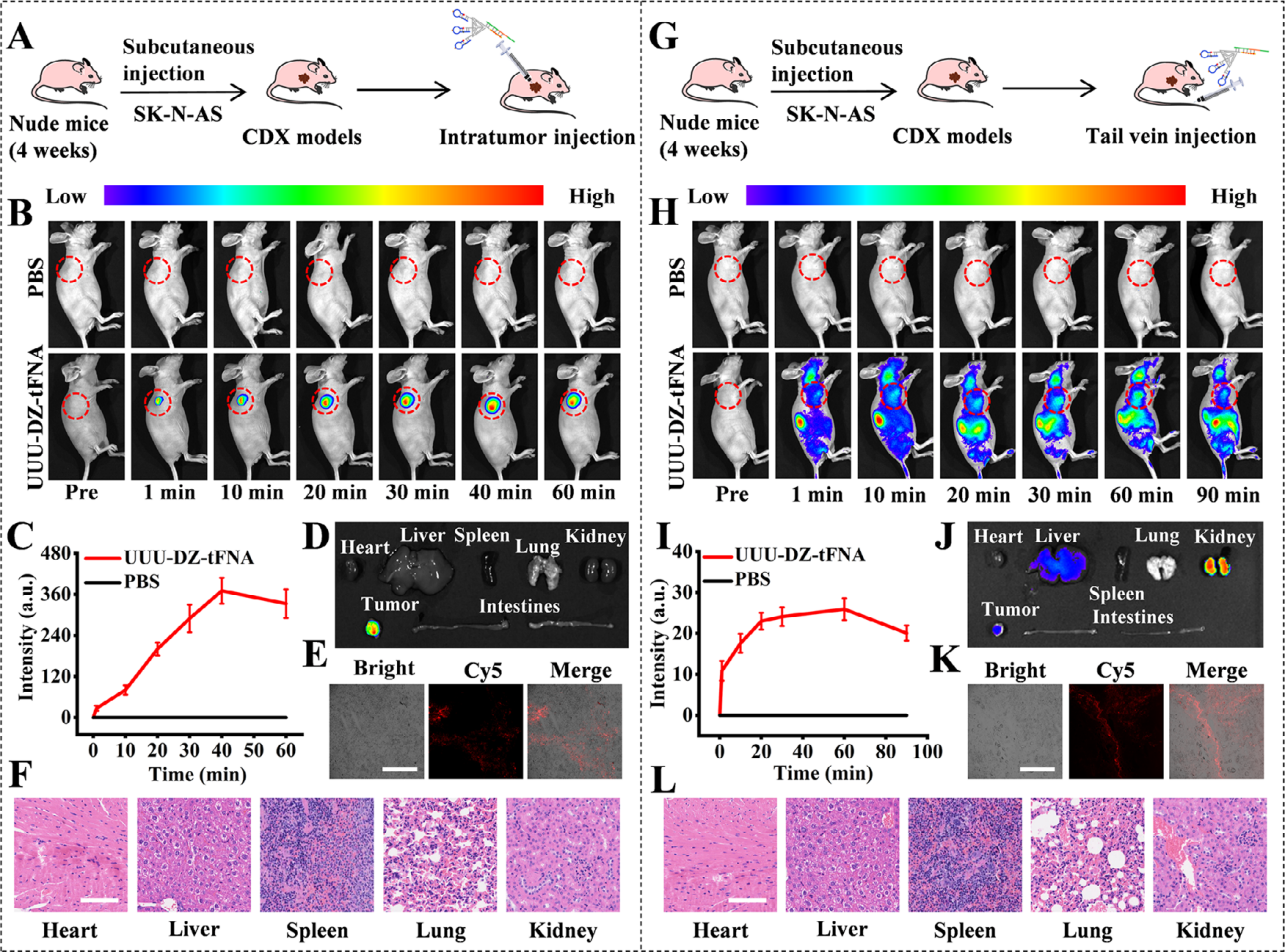
In vivo monitoring of SK‐N‐AS tumor‐bearing mice using UUU‐DZ‐tFNA. The left half (A—F) shows experimentation via intratumor injection of UUU‐DZ‐tFNA in tumor‐bearing mice, and the right half (G—L) shows experimentation via intravenous tail injection of UUU‐DZ‐tFNA in tumor‐bearing mice. (A) Schematic illustration of the experimental procedure for in vivo tumor imaging following intratumoral injection of UUU‐DZ‐tFNA. (B) In vivo fluorescence images of SK‐N‐AS tumor‐bearing mice at 60 min post intratumoral injection of UUU‐DZ‐tFNA; (C) corresponding quantitative fluorescence intensity in the tumor region. (D) Fluorescence images of organs after 60 min of intratumoral injection of UUU‐DZ‐tFNA. (E) Fluorescence images of ex vivo mouse tumor tissue sections. Scale bar: 200 µm. (F) H&E staining images of isolated organs in (B). Scale bar: 100 µm. (G) Schematic illustration of the experimental procedure for in vivo tumor imaging following intravenous tail injection of UUU‐DZ‐tFNA. (H) In vivo fluorescence images of SK‐N‐AS tumor‐bearing mice 90 min after intravenous tail injection of UUU‐DZ‐tFNA, and (I) the corresponding quantitative fluorescence intensity in the tumor region. (J) Fluorescence images of organs after 90 min of intravenous tail injection of UUU‐DZ‐tFNA. (K) Fluorescence images of ex vivo mouse tumor tissue sections. Scale bar: 200 µm. (L) H&E staining images of isolated organs in (H). Scale bar: 100 µm. Data are presented as mean ± standard deviation (*n* = 3).

Based on the outstanding performance of UUU‐DZ‐tFNA for UDG detection in vitro and at the cellular level, we next systematically evaluated its potential for in vivo UDG molecular imaging. A subcutaneous SK‐N‐AS tumor‐bearing mouse model was successfully established at 3 weeks post‐cell implantation, and the in vivo imaging capability of UUU‐DZ‐tFNA was evaluated via intratumoral injection (Figure 5A–F) and tail vein injection (Figure 5G–L). Figure 5A presents a schematic overview of the experimental workflow, including tumor engraftment in nude mice and subsequent intratumoral administration of UUU‐DZ‐tFNA. For intratumoral injection, a fluorescent signal was detectable as early as 1 min post‐UUU‐DZ‐tFNA administration; the signal increased rapidly over time and reached a stable plateau at 40 min. In contrast, no fluorescent signal was detected in mice receiving a phosphate‐buffered saline (PBS) injection, confirming that tissue edema did not interfere with tumor‐specific fluorescent imaging (Figure 5B,C). Sixty minutes after imaging, tumor resection surgery was performed on the mice based on the fluorescence signals in the tumor area. No fluorescence residue was detected at the resection site after tumor removal, confirming the successful resection of subcutaneous tumors mediated by UUU‐DZ‐tFNA (Figure S11). After the maximum fluorescence intensity was reached, the mice were sacrificed, and the major organs (tumor, heart, liver, spleen, lung, kidney, and intestine) were dissected for ex vivo fluorescence imaging. The imaging results revealed that UUU‐DZ‐tFNA was predominantly localized in tumors, corroborating the in vivo imaging data (Figure 5D). Ex vivo frozen sections of tumor tissues from SK‐N‐AS tumor‐bearing mice displayed prominent fluorescent signals (Figure 5E). Hematoxylin and eosin (H&E) staining of the dissected major organs revealed no obvious histological alterations in tissue architecture (Figure 5F), validating the excellent biocompatibility and in vivo safety of UUU‐DZ‐tFNA. In summary, intratumoral injection of UUU‐DZ‐tFNA enables rapid imaging of tumor UDG levels, with no apparent toxicity or side effects observed.

After achieving remarkable results with intratumoral injection, we next evaluated the in vivo imaging capabilities of UUU‐DZ‐tFNA in tumor‐bearing mice upon systemic administration via tail vein injection (Figure 5G–L). Figure 5G depicts the detailed workflow for establishing the tumor‐bearing mouse model and administering UUU‐DZ‐tFNA via tail vein injection. Following tail vein injection, UUU‐DZ‐tFNA exhibited a characteristic in vivo biodistribution profile: robust fluorescent signals rapidly accumulated in the liver and tumor regions within 1 min post‐injection and attained peak intensity at 60 min (Figure 5H,I). This is also consistent with the findings in Figure 2G, confirming the prominent tumor enrichment of UUU‐DZ‐tFNA via the enhanced permeability and retention (EPR) effect. Ex vivo imaging results demonstrated that following tail vein injection, UUU‐DZ‐tFNA was primarily distributed in the tumor, liver, and kidney, consistent with the in vivo imaging data shown in Figure 5H (Figure 5J). Furthermore, the tumor tissue sections exhibited a pronounced fluorescent signal, which corroborated the findings from the in vivo imaging studies shown in Figure 5H (Figure 5K). Serum biochemical analysis revealed no significant fluctuations in alanine aminotransferase (ALT), alkaline phosphatase (ALP), aspartate aminotransferase (AST), and total bilirubin (TBIL) levels before and 90 min after UUU‐DZ‐tFNA administration, indicating that the probe exerted no obvious liver toxic effects in mice (Figure S12). H&E staining of isolated organs, including the heart, liver, spleen, lung, and kidney, revealed no apparent alterations in tissue morphology, suggesting that tail vein injection of UUU‐DZ‐tFNA is unlikely to induce adverse effects on major organs (Figure 5L). In summary, UUU‐DZ‐tFNA enables accurate in situ imaging of UDG in tumor cells via tail vein injection.

### 2.6 Non‐Invasive NB Risk Stratification in Patient‐Derived Xenograft Models using UUU‐DZ‐tFNA

**FIGURE 6:**
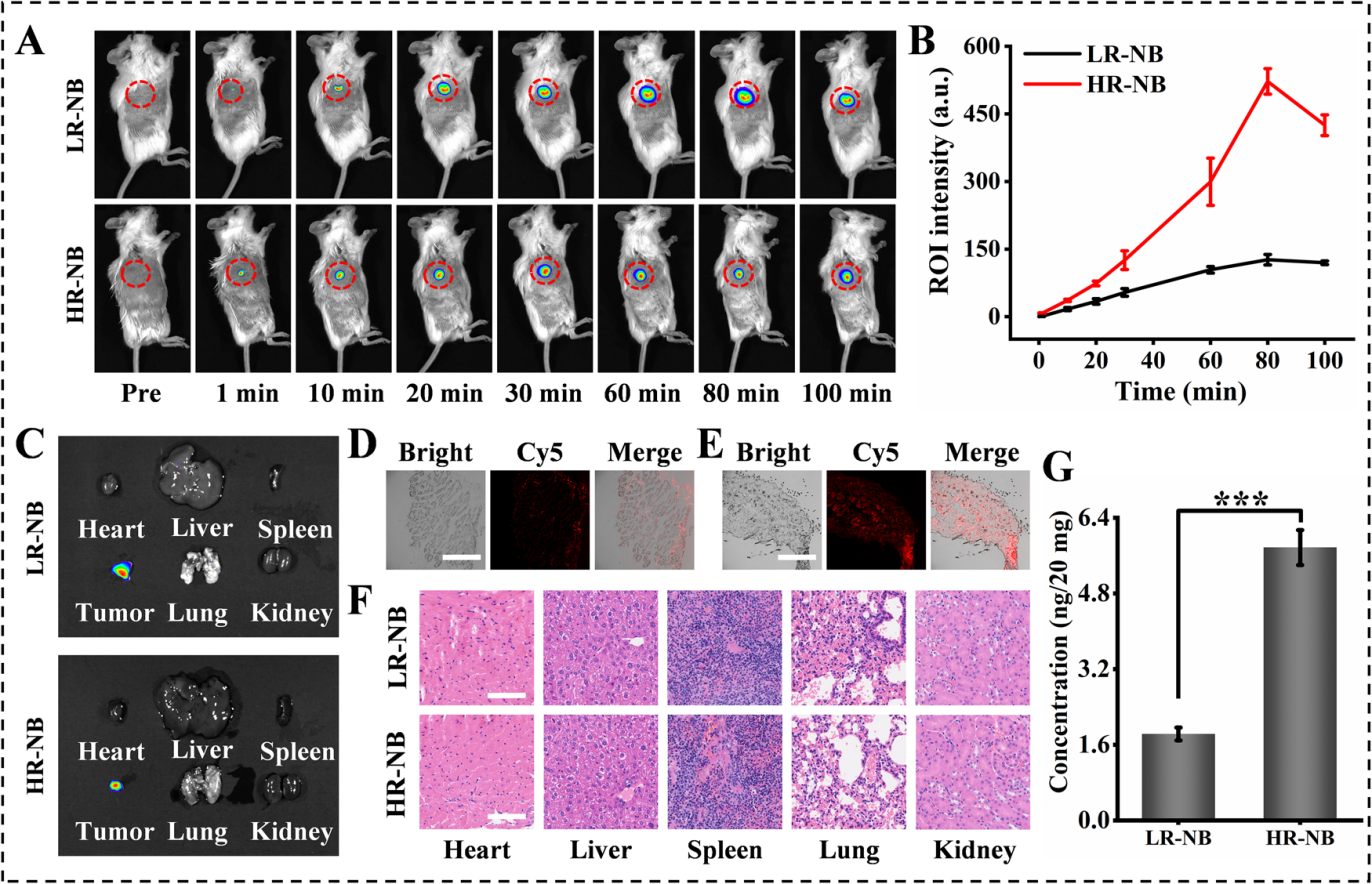
Risk stratification of NB in PDX models with UUU‐DZ‐tFNA. (A) Fluorescence images of LR‐NB and HR‐NB PDX tumors at 100 min post‐intratumoral injection of UUU‐DZ‐tFNA, and (B) the corresponding quantitative fluorescence intensities in the tumor region. (C) Fluorescence images of dissected organs 100 min after intratumoral injection of UUU‐DZ‐tFNA. Fluorescence images of isolated tumor tissues from (D) the LR‐NB and (E) the HR‐NB PDX models. Scale bar: 200 µm. (F) The H&E staining images of isolated organs in (A). Scale bar: 100 µm. (G) Quantitative analysis of tumor UDG expression by ELISA. Data are presented as mean ± SD (*n* = 3). ***P < 0.001.

Precise risk stratification is indispensable for formulating individualized treatment plans and predicting clinical outcomes of NB. Current clinical risk stratification systems integrate multiple indicators, including pre‐operative imaging staging, *MYCN* amplification status, pathological typing, genomic biomarkers, and age at diagnosis [5]. Nevertheless, these evaluation approaches rely on invasive tissue sampling and reflect static disease features, failing to dynamically track the progressive malignant transformation of tumors in a timely manner [64]. Establishing a non‐invasive risk stratification strategy for NB that avoids surgery or tissue biopsy is crucial for improving survival in children with NB [15, 65, 66]. Therefore, to evaluate the value of UDG for risk stratification in NB, we established HR‐NB and low‐risk NB (LR‐NB) patient‐derived xenograft (PDX) models and examined UDG expression levels in tumor tissues in vivo. After intratumoral injection, the imaging capability of UUU‐DZ‐tFNA was evaluated in LR‐NB and HR‐NB PDX models. In vivo fluorescence imaging demonstrated that, within 80 min post‐injection, fluorescence signals in both HR‐NB and LR‐NB PDX models increased progressively over time and peaked at 80 min (Figure 6A,B). At 100 min post‐injection, major organs and tumors were harvested for ex vivo imaging. The results showed that UUU‐DZ‐tFNA was predominantly retained within the tumor, consistent with the in vivo imaging data in Figure 5D (Figure 6C). Frozen section analysis further showed that the UUU‐DZ‐tFNA‐responsive signal was significantly elevated in HR‐NB PDX tumor tissues compared to LR‐NB PDX tumor tissues, consistent with the in vivo imaging results in Figure 6A (Figure 6D,E). H&E staining results corroborated the safety of UUU‐DZ‐tFNA for in vivo monitoring of NB with varying risk grades, as no structural alterations were detected in the resected organs (Figure 6F). ELISA experiments revealed that UDG levels in tumor tissues from HR‐NB PDX mice were significantly higher than those from LR‐NB PDX mice, consistent with the results in Figure 6A (Figure 6G). Together, these findings further confirm that (1) UDG levels can serve as a reliable indicator for risk stratification in NB, and (2) UUU‐DZ‐tFNA has significant application value for non‐invasive risk stratification of NB.

### 2.7 Development of a Non‐Invasive NB Risk Stratification Model using UUU‐DZ‐tFNA

**FIGURE 7:**
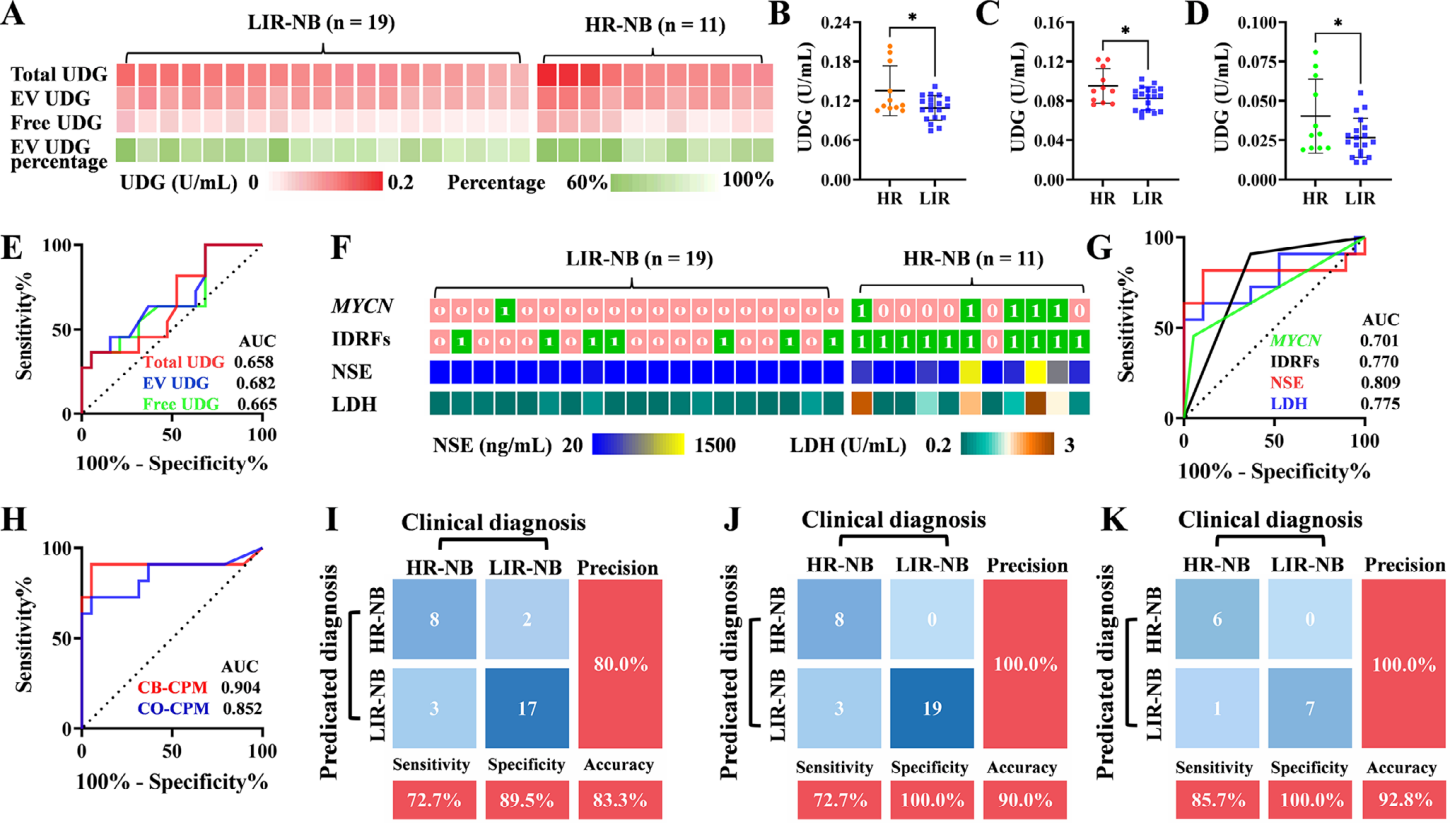
The construction of a non‐invasive risk stratification model for NB using machine learning based on exosomal UDG and clinical multidimensional indicators. (A) Heatmap of total UDG, exosomal UDG, and plasma free UDG levels in plasma from LIR‐NB (*n* = 19) and HR‐NB (*n* = 11) patients, along with the percentage of UDG content localized to exosomes. (B) Total UDG, (C) exosomal UDG, and (D) free UDG levels in plasma samples of children with LIR‐NB and HR‐NB. (E) ROC curves of total, exosomal, and free UDG in plasma for NB risk classification. (F) Heatmap showing the relative abundance of the four clinical indicators identified through screening in LIR‐NB and HR‐NB. (G) ROC curves of *MYCN* amplification status, IDRFs, NSE, and LDH for differentiating LIR‐NB from HR‐NB. (H) ROC curves of CB‐CPM and CO‐CPM for differentiating LIR‐NB from HR‐NB. (I) Confusion matrix analysis shows the diagnostic efficacy of the CO‐CPM model. (J) Training set confusion matrix analysis shows the diagnostic efficacy of the CB‐CPM model. (K) Validation set confusion matrix analysis shows the diagnostic efficacy of the CB‐CPM model. Data are presented as mean ± SD (*n* = 19 for LIR‐NB and *n* = 11 for HR‐NB). *P < 0.05. Abbreviation: EV, exosome.

Protected by a lipid bilayer, plasma exosomal cargoes exhibit high stability, allowing them to dynamically and reliably reflect pathological states and overcome tumor heterogeneity [67, 68]. Consequently, exosomes enable non‐invasive and repeatable detection while avoiding the trauma of tissue biopsy [69, 70, 71, 72]. Therefore, this study systematically evaluated plasma exosomal UDG for NB risk stratification. First, transmission electron microscopy (TEM) confirmed successful exosome extraction (Figure S13A). Meanwhile, SDS‐PAGE followed by Coomassie brilliant blue staining was performed to assess the purity of plasma‐derived exosomes isolated in this work (Figure S13B). Even with increased exosome loading concentrations in two distinct exosome preparations (L1 and L2) derived from the same patient, no prominent band corresponding to 66 kDa albumin or diffuse smearing signals of 200 kDa lipoproteins was detected. The complete absence of these high‐molecular‐weight plasma contaminants verifies the high purity of the extracted exosomes. We further combined proteinase K protection assays with quantitative ELISA to clarify the subvesicular localization of exosomal UDG (Figure S14A). ELISA data revealed that the UDG concentration in the exosome lysate group was markedly higher than that in the intact exosome group, demonstrating that approximately 98.5% of total exosomal UDG resided inside the exosome. Subsequently, immunofluorescence co‐localization imaging was conducted using CD63 as a canonical exosomal membrane marker to visualize the distribution of UDG on or within exosomes (Figure S14B). Obvious spatial overlap between the red UDG channel and the green CD63 channel in the merged image directly confirmed that endogenous UDG derived from NB cells was predominantly encapsulated inside exosomes, with only a minor fraction attached to the exosomal membrane. Exosomes from the same plasma samples were detected using both ELISA kits and UUU‐DZ‐tFNA, revealing a strong correlation between the two methods. This confirmed the reliability of the UUU‐DZ‐tFNA probe for detecting exosomal UDG levels (Figure S15). In addition, to evaluate the value of exosomal UDG for NB risk stratification, we detected total plasma UDG, exosomal UDG, and free UDG using UUU‐DZ‐tFNA. Heatmap analysis revealed that 60.0 – 86.8% of plasma UDG is present in exosomes (Figure 7A). The results showed that UDG levels in HR‐NB patients were higher than those in LR‐NB patients across all three measurements: total plasma UDG, exosomal UDG, and free plasma UDG, with statistically significant differences between the two groups (Figure 7B–D). ROC curve analysis confirmed that exosomal UDG exhibited the optimal diagnostic performance for NB risk stratification (Figure 7E). Therefore, exosomal UDG is expected to serve as a predictive indicator for non‐invasive NB risk stratification.

As shown in Figure 7E, exosomal UDG alone requires improved efficacy for non‐invasive NB risk stratification. Therefore, to enhance clinical applicability, we combined it with multidimensional clinical indicators and developed a machine learning‐based prediction model with high sensitivity and specificity for non‐invasive NB risk stratification. After screening more than ten NB‐related clinical indicators, four parameters with the most significant differences were ultimately selected for subsequent construction of a non‐invasive NB risk stratification model: *MYCN* amplification status (amplification, non‐amplification), IDRFs, NSE, and LDH (Figure 7F). Previously, our research team developed a quantitative method for detecting free *MYCN* copy number in plasma and demonstrated high concordance in *MYCN* amplification status between plasma from NB patients and NB tumor tissues (the clinical gold standard) [73]. Therefore, to achieve non‐invasive NB risk stratification, we used plasma free *MYCN* amplification status as an indicator for constructing the NB risk stratification model in this study. Baseline characteristics of NB patients in the training cohort stratified by LIR‐NB and HR‐NB groups are displayed in Table S3. Single‐indicator prediction results demonstrated that all four non‐invasive indicators exhibited considerable NB risk prediction capacity, with area under the curve (AUC) values greater than 0.7, verifying the effectiveness of each parameter (Figure 7G). Among these, the univariate model based on the serum NSE level showed relatively superior predictive performance, with an AUC of up to 0.81. At the optimal cutoff value, the sensitivity and specificity were 81.8% and 89.5%, respectively, enabling moderate discrimination between HR‐NB and LIR‐NB patients. Therefore, the predictive performance of single indicators is limited and cannot meet the requirements for high‐precision clinical application.

Furthermore, we combined the four clinical non‐invasive indicators, *MYCN* amplification status, IDRFs, NSE, and LDH, to construct a joint clinical prediction model (Clinical‐Only Combined Predictive Model, CO‐CPM). Eleven machine learning classifiers were established, generating eleven classification prediction schemes. Corresponding ROC curves for the eleven algorithms were plotted, and core performance metrics (including AUC, sensitivity, specificity, accuracy, Kappa value, and F1 score) of each model were extracted for comprehensive comparison. Among them, the neural network model presented the best overall performance, with an AUC of 0.852, which was significantly higher than that of the other models (Figure 7H, Table S4, Figure S16). Meanwhile, its core metrics, including sensitivity (72.7%), specificity (94.7%), and accuracy (86.7%), reached optimal levels and could effectively distinguish LIR‐NB patients from HR‐NB ones. The confusion matrix of the model showed that the true positive rate (sensitivity) for HR‐NB patients was 72.7%, and the true negative rate (specificity) for low‐intermediate risk patients was 89.5%, whereas the overall accuracy was only 83.3% (Figure 7I). This suggests that the clinical safety and reliability of the model for non‐invasive prediction of NB risk still need improvement, and further exploration of novel non‐invasive NB risk prediction methods is necessary.

To further enhance the sensitivity and specificity of non‐invasive NB risk stratification, we integrated plasma exosomal UDG with four clinical indicators (*MYCN* amplification status, IDRFs, NSE, and LDH) to form a 5‐feature combined non‐invasive panel. Based on this panel, a Clinical‐Biomarker Combined Predictive Model (CB‐CPM) was constructed using eleven machine learning classifiers, with NB risk stratification as the target variable. Among all models, the neural network algorithm showed optimal performance, with an AUC of 0.904 (95% CI: 0.736 – 1.000) and strong discriminatory ability (Table 1, Figure 7H, and Figure S17). In the training set, the model achieved 72.7% sensitivity, 100.0% specificity, and 90.0% accuracy; in the validation set, it reached 85.7% sensitivity, 100.0% specificity, and 92.8% accuracy (Figure 7J,K). The close agreement of diagnostic efficacy across two independent datasets validates that this neural network holds steady and reliable classification performance for target patient stratification. The AUC of CB‐CPM was 5.2% higher than that of the CO‐CPM, with significantly improved sensitivity and specificity, demonstrating the synergistic value of combining exosomal UDG with multidimensional clinical indicators (Figure 7H). Thus, we innovatively constructed a new method for non‐invasive NB risk prediction, whose advantages are reflected in the following aspects: (1) all indicators in the model can be obtained through non‐invasive or minimally invasive methods, ensuring the non‐invasive nature of the prediction method; (2) the new model combines laboratory indicators with existing clinical indicators, achieving better predictive performance.

**TABLE 1:** Summary of performance data for the CB‐CPM machine learning model.

Model	Best_Sensitivity	Best_Specificity	Best_Cutoff	Best_Youden_Index	ROC	Accuracy	PPV	NPV	Kappa	F1
**glm**	0.8181818	0.78947368	0.272209173	0.60765550	0.7655502	0.8000000	0.6923077	0.8823529	0.5852535	0.7500000
**lda**	0.8181818	0.94736842	0.396229085	0.76555024	0.8421053	0.9000000	0.9000000	0.9000000	0.7804878	0.8571429
**glmnet**	0.9090909	0.89473684	0.373576709	0.80382775	0.8995215	0.9000000	0.8333333	0.9444444	0.7887324	0.8695652
**svmRadial**	0.9090909	0.78947368	0.403766584	0.69856459	0.8516746	0.8333333	0.7142857	0.9375000	0.6606335	0.8000000
**knn**	0.8181818	0.78947368	0.277777778	0.60765550	0.8421053	0.8000000	0.6923077	0.8823529	0.5852535	0.7500000
**rf**	0.8181818	0.57894737	0.190000000	0.39712919	0.7966507	0.6666667	0.5294118	0.8461538	0.3562232	0.6428571
**avNNet**	0.8181818	0.94736842	0.442815437	0.76555024	0.9043062	0.9000000	0.9000000	0.9000000	0.7804878	0.8571429
**nnet**	0.9090909	0.94736842	0.057057722	0.85645933	0.9043062	0.9333333	0.9090909	0.9473684	0.8564593	0.9090909
**naive_bayes**	0.9090909	0.73684211	0.001710646	0.64593301	0.8660287	0.8000000	0.6666667	0.9333333	0.6000000	0.7692308
**fda**	0.9090909	0.52631579	0.073958349	0.43540670	0.7224880	0.6666667	0.5263158	0.9090909	0.3775934	0.6666667
**rpart**	0.8181818	0.78947368	0.130252101	0.60765550	0.7703349	0.8000000	0.6923077	0.8823529	0.5852535	0.7500000

*Abbreviations*: glm, Generalized Linear Model; lda, Linear Discriminant Analysis; glmnet, Generalized Linear Model via Regularization; svmRadial, Support Vector Machine with Radial Basis Function kernel; knn, K‐Nearest Neighbors; rf, Random Forest; avNNet, Average Neural Network; nnet, Neural Network; naive_bayes, Naive Bayes Classifier; fda, Flexible Discriminant Analysis; rpart, Recursive Partitioning and Regression Trees.

### 2.8 UUU‐DZ‐tFNA for In Vivo Monitoring of NB Drug Resistance

**FIGURE 8:**
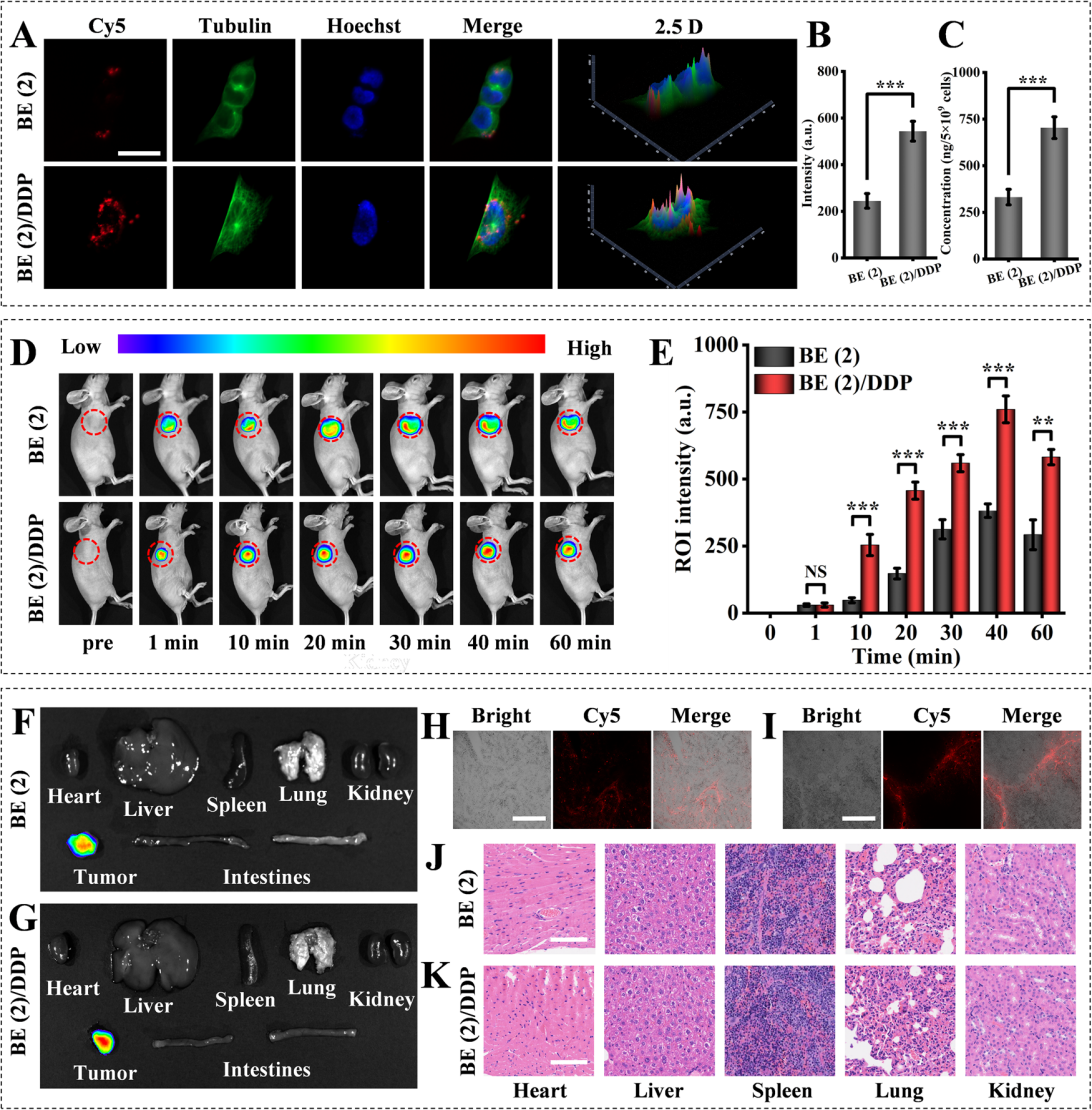
UUU‐DZ‐tFNA for monitoring NB drug resistance. (A) Fluorescence microscopy images of SK‐N‐BE (2)/DDP and SK‐N‐BE (2) cells treated with UUU‐DZ‐tFNA. (B) Corresponding quantitative Cy5 fluorescence intensity histogram for (A). Scale bar: 40 µm. (C) Cytoplasmic UDG levels in SK‐N‐BE (2)/DDP and SK‐N‐BE (2) cells quantified using the UDG ELISA kit. (D) In vivo fluorescence images of SK‐N‐BE (2)/DDP and SK‐N‐BE (2) tumor‐bearing mice at 60 min post‐intratumoral injection, and (E) the corresponding tumor fluorescence intensity. (F, G) Fluorescence images of tumors and organs isolated 60 min after intratumoral injection of UUU‐DZ‐tFNA. Ex vivo fluorescence images of tumor tissues from (H) SK‐N‐BE (2) and (I) SK‐N‐BE (2)/DDP tumor‐bearing mice. Scale bar: 200 µm. H&E staining of ex vivo organs from (J) SK‐N‐BE (2) tumor‐bearing mice and (K) SK‐N‐BE (2)/DDP tumor‐bearing mice. Scale bar: 100 µm. Data are presented as mean ± SD (*n* = 3). **P < 0.01; ***P < 0.001. NS: no significant difference.

Despite immunotherapy and targeted therapy emerging as popular research directions, chemotherapy remains the dominant clinical strategy to suppress tumor progression, and perioperative adjuvant chemotherapy serves as a core component of HR‐NB standard treatment [10]. Most children with high‐risk NB achieve favorable initial responses to chemotherapy; nevertheless, over 60% of them develop acquired multidrug resistance, which triggers tumor relapse and/or metastasis and ultimately leads to patient death [8, 74, 75]. Early identification of emerging drug resistance is essential to block tumor progression and prevent metastatic spread. Currently, the clinical assessment of drug resistance in HR‐NB relies on tumor recurrence as the main criterion, which is characterized by considerable delay and poor diagnostic timeliness. When resistance is detected, systemic metastasis has often already occurred, leading to the loss of the optimal treatment window [15, 65]. Thus, the 5‐year survival rate for children with HR‐NB is still approximately 48% [76]. Therefore, timely monitoring of drug resistance in HR‐NB to guide precision medicine remains an urgent and important challenge. This study is the first to systematically investigate the value of UUU‐DZ‐tFNA in monitoring NB resistance by targeting UDG, aiming to provide a new strategy for NB resistance monitoring. At the cellular level, UDG expression in NB drug‐resistant cells (SK‐N‐BE (2)/DDP) and parental SK‐N‐BE (2) cells was detected using UUU‐DZ‐tFNA. The results showed that the UUU‐DZ‐tFNA signal (Cy5) was significantly higher in SK‐N‐BE (2)/DDP cells than in SK‐N‐BE (2) cells (Figure 8A,B). To further validate these findings, we quantified cytoplasmic UDG expression in SK‐N‐BE (2)/DDP and SK‐N‐BE (2) cells using an ELISA kit. The results showed that UDG levels in the cytoplasm of SK‐N‐BE (2)/DDP cells were significantly higher than those in SK‐N‐BE (2) cells (Figure 8C). These results indicate that UDG is significantly overexpressed in the cytoplasm of SK‐N‐BE (2)/DDP cells, and that UUU‐DZ‐tFNA is a reliable tool for detecting cytoplasmic UDG.

Then, UUU‐DZ‐tFNA was administered via intratumoral injection into SK‐N‐BE (2)/DDP tumor‐bearing mice or parental SK‐N‐BE (2) tumor‐bearing mice (Figure 8D). In vivo fluorescence imaging revealed a rapid, robust, and sustained signal increase specifically in SK‐N‐BE (2)/DDP tumors—significantly exceeding that in SK‐N‐BE (2) controls beginning at 10 min post‐injection, with maximal intergroup divergence observed at 40 min (Figure 8D,E). Ex vivo fluorescence imaging conducted 60 min post‐injection confirmed that tumor tissues from SK‐N‐BE (2)/DDP tumor‐bearing mice exhibited higher fluorescence signals than those from parental SK‐N‐BE (2) tumor‐bearing mice (Figure 8F,G). In addition, tumor tissue sections showed stronger fluorescence signals in SK‐N‐BE (2)/DDP tumors than in SK‐N‐BE (2) tumors, consistent with the in vivo imaging results in Figure 8D and the ELISA results in Figure 1D (Figure 8H,I). H&E‐stained images of isolated organs showed unaltered tissue structure, suggesting that intratumoral injection of UUU‐DZ‐tFNA is safe (Figure 8J,K). In summary: (i) cytoplasmic UDG expression is significantly upregulated in cisplatin‐resistant NB cells; (ii) UUU‐DZ‐tFNA enables in vivo monitoring of NB drug resistance.

## 3 Conclusions

In summary, we have developed an engineered allosteric biosensor characterized by high sensitivity and specificity for in situ UDG imaging. UUU‐DZ‐tFNA offers several notable advantages. First, DNA sequences partially complementary to the DNAzyme structural domain were strictly screened and verified as the locking chain. This chain effectively prevents the allosteric function of the DNAzyme, thereby minimizing non‐specific signal leakage. Second, the rationally designed dU modification sites serve as precise molecular triggers: their selective excision by UDG destabilizes the DNAzyme–lock strand duplex. This allosteric change releases the active DNAzyme, which then catalyzes cleavage at the rA site within the HP2 (S7), initiating a robust signal amplification cascade. Third, UUU‐DZ‐tFNA not only enables in vivo imaging of UDG in tumor cells but also facilitates the establishment of a new method for non‐invasive NB risk stratification and in vivo NB drug resistance monitoring. Nevertheless, this work has two inherent limitations. First, UUU‐DZ‐tFNA is a fluorescent probe featuring limited tissue penetration, which precludes its use for in vivo orthotopic tumor detection. Second, all clinical specimens were collected from a single center; multi‐center datasets will be incorporated in follow‐up investigations to improve the model's generalizability. In conclusion, UUU‐DZ‐tFNA achieves in situ UDG detection across multiple biological levels and facilitates risk stratification and drug resistance monitoring of NB.

## 4 Materials and Methods

### 4.1 Materials

APE1 and UGI were obtained from New England Biolabs (Ipswich, Massachusetts, USA). UDG, deoxyribonuclease I (DNase I), and ribonuclease A (RNase A) were obtained from Thermo Fisher Scientific (Waltham, Massachusetts, USA). All DNA sequences (Table S1) and the cytoplasmic protein extraction kit were sourced from Sangon Biotech Co., Ltd. (Shanghai, China). The UDG ELISA kit was sourced from Enzyme‐Linked Immunoassays (Wuhan, China). The CCK‐8 assay kit, proteinase K, and exosome extraction kit were obtained from Nanjing Vazyme Biotech Co., Ltd. (Nanjing, China). The Tubulin‐Tracker Green Staining Kit and the Coomassie Blue Ultra‐Fast Staining Solution were sourced from Beyotime Biotechnology (Shanghai, China). The anti‐UDG rabbit monoclonal antibody, the anti‐CD63 mouse monoclonal antibody, the GF555‐conjugated goat anti‐rabbit IgG, and the BF488‐conjugated goat anti‐mouse IgG were purchased from Shanghai Epizyme Biomedical Technology Co., Ltd. (Shanghai, China). Four‐week‐old female BALB/c nude mice and four‐week‐old female NOG mice were purchased from Beijing Vital River Laboratory Animal Technology Co., Ltd. (Beijing, China). Matrigel was obtained from BD Biosciences (Franklin Lakes, New Jersey, USA). Unless otherwise explicitly stated, all reagents were procured from commercial suppliers and used directly without further purification.

### 4.2 Clinical Sample Collection and UDG Quantification

All clinical samples used in this study were acquired with the approval of the Ethics Committee of Henan Children's Hospital, Zhengzhou Children's Hospital (No. 2025‐1IT‐0047‐001). Written informed consent was obtained from the guardians of all pediatric participants prior to sample collection, and all experimental procedures strictly adhered to relevant ethical guidelines and regulatory mandates. From October 2023 to January 2025, a total of 50 plasma specimens were collected from histopathologically diagnosed NB patients, including paired baseline specimens before chemotherapy and specimens after multiple rounds of chemotherapy derived from the same patients. Among them, 30 pretreatment specimens were assigned to the training set. An independent validation cohort consisting of 14 plasma samples was further enrolled from newly diagnosed NB patients between February 2025 and April 2026 for external verification of the predictive model.

### 4.3 Synthesis and Characterization of UUU‐DZ‐tFNA

Initially, 1 µL of each of the four single‐stranded DNA oligonucleotides (S1 – S4; 100 µM) was mixed with 18 µL of TEM buffer (20 mM Tris‐HCl, 2 mM EDTA, 12.5 mM MgCl_2_·6H_2_O) and 38 µL of DEPC‐treated water (pH 7.4). The mixture was heated at 95°C for 10 min to induce denaturation, followed by slowing down to 25°C at a rate of −1°C per min to facilitate self‐assembly into tFNA. Subsequently, 1 µL of S5 (100 µM) and 1.2 µL of S6 (100 µM) were combined with 3 µL of TEM buffer and 4.8 µL of DEPC‐treated water. This mixture was subjected to the same denaturation protocol (95°C for 10 min) and then rapidly cooled to 25°C at −1°C per minute to form the S5‐S6 duplex. Thereafter, 3 µL of S7 (100 µM) was mixed with 9 µL of TEM buffer and 18 µL of DEPC‐treated water, followed by heating at 95°C for 10 min and gradual cooling to 25°C at −1°C per minute to prepare HP2. Finally, tFNA, the S5‐S6 duplex, and HP2 were co‐incubated at 37°C for 30 min to allow hybridization, yielding the UUU‐DZ‐tFNA nanostructure. Successful assembly of UUU‐DZ‐tFNA was verified by an AGE run at 120 V for 50 min. Additionally, the morphology of UUU‐DZ‐tFNA was characterized by AFM.

### 4.4 In Vitro Detection of UDG by UUU‐DZ‐tFNA

UUU‐DZ‐tFNA (100 µm) was diluted to a final concentration of 100 nm in a buffer containing 20 mm Tris‐HCl, 2 mm EDTA, and 12.5 mm MgCl_2_·6H_2_O (pH 7.4), with the total volume adjusted to 100 µL. The diluted UUU‐DZ‐tFNA solution was then mixed with optimized concentrations of UDG (1 U/mL) and Zn^2+^ (20 µm) and incubated at 37°C for 3 h. The reaction mixture was subsequently analyzed by AGE and capillary electrophoresis. Fluorescence spectroscopy (excitation wavelength: 655 nm) was performed using a microplate reader (SpectraMax i3x) to obtain kinetic profiles and full fluorescence spectra. For the UDG sensitivity assay, UUU‐DZ‐tFNA was added to UDG solutions at concentrations ranging from 0 to 5 U/mL (0, 0.05, 0.5, 1, 0.01, 0.1, 0.5, 1, 2, and 5 U/mL), with simultaneous supplementation of 20 µM Zn^2+^, followed by incubation at 37 °C for 3 h. To assess the specificity of UUU‐DZ‐tFNA for UDG detection, 0.1 U/mL of individual nucleases—including UDG, DNase I, RNase A, and APE1—was added to separate reaction systems containing 100 nM UUU‐DZ‐tFNA and 20 µM Zn^2+^. After incubation at 37°C for 3 h, the fluorescence intensity in each sample was measured.

### 4.5 Stability of UUU‐DZ‐tFNA

The thermal stability of UUU‐DZ‐tFNA was evaluated by monitoring AGE results over a 24‐h incubation period at 4°C, 25°C, and 37°C using a concentration of 100 nm. For serum stability assessment, UUU‐DZ‐tFNA (100 nM) was incubated in fetal bovine serum (FBS) at concentrations of 10%, and AGE analysis was performed at 0, 4, 8, 16, and 24 h to monitor structural integrity.

### 4.6 Cell Culture and Cell Cytotoxicity Test

Human embryonic kidney epithelial cells (293T), human NB cell lines (SK‐N‐BE (2) and SK‐N‐AS), non‐small cell lung cancer cells (A549), and a cisplatin‐resistant NB cell line (SK‐N‐BE (2)/DDP, self‐established) were cultured in Dulbecco's Modified Eagle Medium (DMEM) supplemented with 10% FBS and 1% penicillin–streptomycin under standard conditions (37°C, 5% CO_2_, humidified atmosphere). The SK‐N‐BE (2)/DDP cell line was established by exposing SK‐N‐BE (2) cells to stepwise increasing concentrations of cis‐diamminedichloroplatinum (DDP), ranging from 2 to 8 µm. Primary human neurons and neural crest cells are impractical for extensive in vitro validation due to limited accessibility, lack of proliferative capacity, and cumbersome stem cell differentiation procedures with unstable phenotypes. Thus, A549 and 293T cell lines were adopted as alternative controls. A549 was used to verify the broad‐spectrum UDG detection capacity of UUU‐DZ‐tFNA across tumor types, while stably cultured 293T cells served as a non‐tumor reference to evaluate basal probe uptake and background signals. To evaluate the cytotoxic effect of UUU‐DZ‐tFNA on these cell lines, CCK‐8 assays were performed. Cells were seeded in 96‐well plates at a density of 1 × 10^4^ cells per well and allowed to adhere for 12 h. Subsequently, cells were treated with complete medium containing varying concentrations of UUU‐DZ‐tFNA (0, 50, 100, and 200 nm) for 8 h. After treatment, the wells were washed three times with PBS to remove unbound nanostructures. Then, 100 µL of fresh medium supplemented with 10% CCK‐8 reagent was added to each well. Following a 4‐h incubation, absorbance was measured at 450 nm using a microplate reader (Multiskan Go).

### 4.7 Evaluation of Cellular Uptake Ability of UUU‐DZ‐tFNA

SK‐N‐BE (2) cells were seeded in 20 mm glass‐bottom confocal dishes at a density of 5 × 10^3^ cells per dish and cultured for 12 h. Following treatment with UUU‐DZ‐tFNA (200 nM) for varying durations (2, 4, 6, and 8 h), the cells were washed with PBS and imaged under a 40× objective lens using an Axiovert 5 inverted fluorescence microscope (Zeiss, Germany).

### 4.8 Cellular UDG Expression Detected by UUU‐DZ‐tFNA

293T, SK‐N‐BE (2), SK‐N‐AS, A549, and BE (2)/DDP cells were seeded in 20 mm glass‐bottom confocal dishes and incubated at 37°C and 5% CO_2_ for 12 h to facilitate cell adhesion to the dish wall. Following serum starvation, the cells were initially treated with a 50 µM Zn^2+^ solution in serum‐free DMEM medium for 30 min. Subsequently, they were treated with a 200 nM UUU‐DZ‐tFNA complex in serum‐free DMEM medium for 6 h [28, 77]. Similarly, the two control probes (nUUU‐DZ‐tFNA and nrA‐UUU‐DZ‐tFNA) were administered at the identical concentration and incubation duration to maintain consistent experimental conditions. Unbound complexes were then washed three times with 0.1 M PBS (pH 7.4). The cytoskeleton and nucleus were stained with Tubulin and Hoechst, respectively. Cell images were observed using an Axiovert 5 inverted fluorescence microscope (Zeiss, Germany). In addition, cells were pre‐incubated with the UDG inhibitor (UGI, 2 U/mL stock solution in 1 × UDG Reaction Buffer) for 30 min. Subsequently, UUU‐DZ‐tFNA (200 nM) was added to the cells for an additional 6 h of incubation. Finally, the cells were washed three times with PBS to remove uninternalized UUU‐DZ‐tFNA and stained with Tubulin and Hoechst for fluorescence imaging.

### 4.9 Flow Cytometric Fluorescence Analysis

293T, A549, SK‐N‐BE (2), SK‐N‐AS, and SK‐N‐BE (2)/DDP cells were seeded in T25 culture flasks. Once cell confluence reached 90%, the culture medium was replaced with serum‐free DMEM, and 200 nM UUU‐DZ‐tFNA was added. Cells were harvested by lysis with 0.25% hyaluronidase–EDTA, followed by centrifugation. The cells were then resuspended in PBS for flow cytometry analysis.

### 4.10 UUU‐DZ‐tFNA for Detection of UDG In Vivo

All animal experiments strictly adhered to the Animal Care Guidelines (Protocol No. 2025‐KY‐0011‐001). Specific‐pathogen‐free (SPF) female BALB/c nude mice, aged 3 – 4 weeks and weighing 14 – 29 g, were procured from Beijing Victron Life Science Co., Ltd. (Beijing, China) and housed in an SPF animal facility. Environmental conditions were as follows: temperature maintained at 22°C ± 2°C, humidity at 50 ± 5%, a 12‐h light‐dark cycle, and ad libitum access to food and water. The acclimation period prior to the experiment was 14 days. Four‐week‐old female BALB/c nude mice were subcutaneously inoculated with 6 × 10^6^ SK‐N‐BE (2), SK‐N‐AS, or SK‐N‐BE (2)/DDP cells in a 50 µL mixture of PBS and Matrigel (1:1, v/v) in the right scapular region. Once the tumor volume reached approximately 300 mm^3^, intratumoral injections of either PBS or UUU‐DZ‐tFNA (100 µL, 1 µM) were administered to the tumor‐bearing mice [46, 78]. Another cohort of mice received intravenous injections of the same dose of PBS or UUU‐DZ‐tFNA (100 µL, 1 µM) via the tail vein. The AniView600 in vivo imaging system (excitation wavelength: 625 nm; emission wavelength: 680 nm) was used to perform fluorescence imaging at various time points. Serum samples were harvested by abdominal puncture prior to and 90 min following UUU‐DZ‐tFNA injection for quantification of liver function biochemical markers, including ALT, ALP, AST, and TBIL. Immediately after euthanasia, tumor tissues and major organs were harvested for ex vivo imaging. The excised tumors were sectioned into 3 – 4 µm thick slices and imaged using a Leica fluorescence microscope. Simultaneously, tissues, including the heart, liver, spleen, lung, and kidney were collected for preparation of paraffin‐embedded sections. The sections were stained with H&E to assess tissue structural integrity.

### 4.11 PDX Model Construction and In Vivo Imaging

All PDX models employed in this study were derived from primary NB tumors with clear clinical risk stratification strictly based on the COG risk classification system [5]. SPF female NOG mice, aged 3 – 4 weeks and weighing 12 – 14 g, were procured from Beijing Weitong Biotechnology Co., Ltd. and housed in SPF individually ventilated cages under conditions of 22°C ± 2°C, 55 ± 5% relative humidity, and a 12‐h light/dark cycle. After 7 days of acclimation, fresh NB tissues surgically excised from patients with pathologically confirmed NB were rinsed three times with PBS containing sterile antibiotics to remove blood, necrotic tissue, and connective tissue. The tissues were then cut into 1 – 2 mm^3^ blocks, disinfected with iodophor, and subcutaneously implanted into the mice using a needle. Two to three mice were housed per cage, and their mental state, feeding behavior, and wound healing were monitored daily. Starting on day 7 post‐transplantation, tumor growth was measured every 3 days using a vernier caliper, and tumor volume was calculated as V = (length × width^2^) / 2. When tumor volume reached 50 – 100 mm^3^, mice were euthanized by decapitation, and tumor tissues were collected under sterile conditions, cut into 1 – 2 mm^3^ pieces, and re‐transplanted into new NOG mice for passaging (designated as the F1 generation; F1 – F3 generations were used for subsequent experiments). For in vivo imaging, UUU‐DZ‐tFNA (10 µL, 1 µM) was injected intratumorally when the tumor volume reached 50 – 100 mm^3^. At 1, 10, 20, 30, 60, 80, and 100 min post‐injection, images were acquired using an AniView600 in vivo imaging system (excitation: 625 nm; emission: 680 nm). Tumors and major organs were collected for ex vivo imaging. Tumor tissues were sectioned into 3 – 4 µm thick slices and imaged using a Leica fluorescence microscope.

### 4.12 SDS‐PAGE and Coomassie Brilliant Blue Staining

Exosome pellets were isolated from plasma using an exosome extraction kit and fully resuspended in RIPA lysis buffer supplemented with phenylmethylsulfonyl fluoride (PMSF) and a protease inhibitor cocktail. The mixture was incubated on ice for 30 min with repeated pipetting, then centrifuged at 12 000 g for 30 min. The supernatant containing total exosomal protein was collected, and protein concentration was determined via a BCA protein assay kit. 10 µg and 20 µg of exosome proteins were respectively subjected to denaturation in a 95°C water bath and then separated by SDS‐PAGE. After electrophoresis, the gel was stained with Coomassie Brilliant Blue solution on a shaker for 30 min and destained with ultrapure water. Finally, gel images were captured for subsequent analysis.

### 4.13 Proteinase K Protection Assay

Exosome pellets isolated from the plasma of identical patients were resuspended in 300 µL PBS and equally divided into three groups: the exosomes group, exosomes + proteinase K (PK) group, and exosome lysis group. For the exosomes + PK group, exosome suspensions were treated with 20 µg/mL proteinase K at 37°C for 60 min, followed by the addition of 5 mM PMSF for 10 min at room temperature to terminate protein digestion. For the exosome lysis group, exosome samples were fully lysed with sufficient RIPA lysis buffer supplemented with a protease inhibitor cocktail and PMSF, and incubated on ice for 30 min with repeated pipetting during the lysis period. The exosomes group was supplemented with an appropriate volume of PBS to ensure a consistent final volume with the other two groups. After the completion of all treatments, the UDG concentration of each group was detected using a UDG ELISA kit.

### 4.14 Exosome Immunofluorescence Analysis

Glass coverslips were pre‐coated with 0.1 mg/mL poly‐L‐lysine for 30 min, rinsed with PBS, and air‐dried under sterile conditions. Purified exosomes were diluted with PBS to a concentration ranging from 1 × 10^9^ to 1 × 10^10^ particles/mL. A volume of 20 – 30 µL of exosome suspension was dropped onto each coverslip, followed by incubation in a humidified chamber at room temperature for 1 h. Subsequently, samples were fixed with paraformaldehyde and washed thoroughly. The samples were permeabilized with 0.1% Triton X‐100 for 10 min and then blocked with 0.5% goat serum at 37°C for 1 h. Primary antibodies against UDG and CD63 were diluted at a ratio of 1:100, applied to exosome samples, and incubated overnight at 4°C. After extensive washing the next day, GF555‐ and BF488‐conjugated IgG secondary antibodies (diluted 1:1000) were added and incubated at room temperature for 1 h. Following another thorough washing step, the samples were mounted with anti‐fade mounting medium and sealed with neutral glycerol. All prepared samples were visualized under a polar‐SIM polarization structured illumination super‐resolution microscopy system.

### 4.15 Exosomes UDG Detection Using UUU‐DZ‐tFNA

Plasma exosomes were extracted using the plasma exosome isolation kit (R603; Nanjing Novozyme Biotechnology Co., Ltd., Nanjing, China). A 90 µL aliquot of plasma from NB patients was mixed with 10 µL of reaction buffer containing 1 µM UUU‐DZ‐tFNA and incubated at 37°C for 3 h. The emission spectrum was then recorded using a microplate reader to quantify total UDG activity in plasma. Plasma exosomes isolated from NB patient plasma (90 µL, resuspended in PBS) were mixed with 10 µL of reaction buffer containing 1 µM UUU‐DZ‐tFNA and incubated at 37°C for 3 h. The emission spectrum was recorded using a microplate reader to quantify UDG activity in plasma exosomes. Free UDG activity in plasma was calculated as the difference between total plasma UDG activity and exosomal UDG activity.

### 4.16 Detection Methods for Machine Learning‐Based Predictive Indicators

For *MYCN* amplification status, our team established a quantitative assay for plasma cell‐free *MYCN* copy number measurement in prior research [73], which exhibited high concordance with tissue FISH, the clinical gold standard. To realize non‐invasive NB risk stratification, we adopted plasma cell‐free *MYCN* status rather than invasive tumor tissue detection as a predictive marker. IDRFs were assessed via pretreatment contrast‐enhanced CT or whole‐body MIBG imaging per INRG criteria; all images were reviewed by specialized radiologists, and cases with any high‐risk anatomical features were identified as IDRF‐positive. Fasting venous blood was collected to quantify serum markers: NSE was tested on a Cobas e801 chemiluminescence analyzer (Roche, Germany), and LDH was measured with an AU5800 biochemical analyzer (Beckman, USA).

### 4.17 Statistical Analysis

All statistical analyses presented in tables were performed using SPSS Statistics 21 software. Statistical tests for data shown in figures were conducted using GraphPad Prism 9 and Origin 2024 software. Machine learning model construction was performed within the R programming environment (R 4.5.2). All statistical tests were two‐sided with an alpha value set at 0.05. Categorical variables were compared using Fisher's exact test. Normally distributed quantitative data were presented as mean ± standard deviation (SD) and compared by independent‐samples *t*‐test. Skewed quantitative indicators were expressed as median (interquartile range) and analyzed via the Mann‐Whitney U test. Comparisons among three or more groups were performed using one‐way analysis of variance (ANOVA). Statistical significance was denoted by asterisks: *P < 0.05; **P < 0.01; ***P < 0.001; n.s. indicates no significant difference.

## Author Contributions

Y.L. and Y.Z. performed the experiments and wrote the original manuscript. X.Z. advised on the research direction and designed the methodology. M.Z. and F.Z. assisted with the experiments and provided experimental methods. J.S. and Y.Y. performed data curation and formal analysis. K.L. participated in the validation experiments. P.W., S.L., and W.Z. supervised this study and revised the manuscript.

## Funding

This work was funded by the National Natural Science Foundation of China (32201237), Scientific and Technological Projects of Henan Province (242102310383, 262102311041, 262102311123, 262102310053), Henan Medical Science and Technology Program (SBGJ202403052, SBGJ202502095, LHGJ20250596, LHGJ20250605, LHGJ20230583), Zhengzhou Outstanding Young Scientific and Technological Talents (Doctor) Training Project (2024046). Zhengzhou Zhongrui Foreign Expert Workstation for Precision Diagnosis and Treatment of Neuroblastoma (ZZGZS2024006).

## Ethics Approvals

Ethical approval was obtained from the Laboratory Animal Ethics Committee of Henan Shuangyun Biotechnology Co., Ltd. (approval number: SYLS2025063). For human‐related retrospective analysis involving NB patient samples, the study protocol was reviewed and approved by the Institutional Review Board of Henan Children's Hospital, Zhengzhou Children's Hospital (approval number: 2025‐IIT‐0047‐001).

## Conflicts of Interest

The authors declare no conflicts of interest.

## Supporting information




**Supporting File**: advs77924‐sup‐0001‐SuppMat.docx.

## Data Availability

The data presented in this study are available upon reasonable request from the corresponding author.
