# Supplementary material for: Engineered Allosteric Biosensor for In Situ DNA Glycosylase Detection in Neuroblastoma Risk Stratification and Drug Resistance Monitoring
Source: Adv Sci (Weinh). 2026 Sep 27:e77924. Online ahead of print. doi: 10.1002/advs.77924 (PMC13616298; doi:10.1002/advs.77924)
Supplement: Supplementary file 1 [file ADVS-9999-e77924-s001.docx]

**Supporting Information**

**Engineered Allosteric Biosensor for *In Situ* DNA Glycosylase Detection in Neuroblastoma Risk Stratification and Drug Resistance Monitoring**

Yaqing Lu^1, #^, Yingyu Zhang^2,3 #^, Xianwei Zhang^1, #^, Mengxin Zhang^1, 5^, Fei Zhang^1^, Jushan Sun^1^, Yahui Yang^1^, Kangbo Liu^4^, Peng Wang^6, *^, Shuying Luo^1, *^, Wancun Zhang^1, *^

^1^Health Commission of Henan Province Key Laboratory for Precision Diagnosis and Treatment of Pediatric Tumor, Children's Hospital Affiliated to Zhengzhou University, Zhengzhou, 450018, China

^2^Henan Key Laboratory of Rare Diseases, Endocrinology and Metabolism Center, The First Affiliated Hospital, and College of Clinical Medicine of Henan University of Science and Technology, Luoyang, 471003, China

^3^State Key Laboratory of Light Superalloys, Henan University of Science and Technology, Luoyang 471023, China

^4^NMPA Key Laboratory for Quality Evaluation of Medical Protective and Implant Devices, Henan Institute for Drug and Medical Device Inspection (Henan Vaccine Issuance Center), Zhengzhou, 450018, China

^5^College of Public Health, Zhengzhou University, Zhengzhou 450001, P. R. China

^6^Department of Biomedical Engineering, School of Engineering, China Pharmaceutical University, Nanjing, 210009, China

^#^These authors contributed equally to this article. ^*^Corresponding authors: Wancun Zhang (zhangwancun@126.com); Shuying Luo [(shyluo@163.com);](mailto:(shyluo@163.com);) Peng Wang (wangpeng@cpu.edu.cn). Tel: + 86-373-85515773; Fax: +86-373-85515773

**Figure S1** (A - B) Capillary electrophoresis results demonstrated the successful preparation of UUU-DZ-tFNA. L1, S1; L2, S1 + S2; L3, S1 + S2 + S3; L4, S1 + S2 + S3 + S4; L5, S5 + S6; L6, S7; L7, UUU-DZ-tFNA.

**Figure S2** Stability analysis of UUU-DZ-tFNA by AGE.

**Figure S3** The conformational activation mechanism of the DNA enzyme triggered by UDG was verified through 1.5% AGE. M, DNA marker; L1, S5; L2, S5-S6; L3, S5-S6 + UDG; L4, HP2; L5, S5-S6 + HP2; L6, S5-S6 + HP2 + UDG.

**Figure S4** (A - B) Capillary electrophoresis analysis showing the response of UUU-DZ-tFNA to UDG.

**Figure S5** (A) Reaction kinetics of the UUU-DZ-tFNA response to UDG. (B) Reaction kinetics of the UUU-DZ-tFNA response to UDG under different concentrations of HP2 conditions.

**Figure S6** Corresponding calibration curves of UDG concentration versus fluorescence intensity for **Figure 3C**.

**Figure S7** Cytotoxicity of UGI at various concentrations (0, 0.5, 1, and 2 U/mL) in SK-N-BE (2) and SK-N-AS cells, showing no significant differences. Error bars represent the mean ± standard deviation of three independent experiments.

**Figure S8** (A) Optimization of UUU-DZ-tFNA (200 nM) incubation time in SK-N-BE (2) cells. (B) The corresponding fluorescence intensity (Cy5) histograms in (A). Data are means ± SD (n = 3). **P* < 0.05; ****P* < 0.001. NS: no significant difference; BE (2), SK-N-BE (2).

**Figure S9** Cytotoxicity of UUU-DZ-tFNA at various concentrations (0, 50, 100, and 200 nM) in 293T, SK-N-AS, and SK-N-BE (2) cells, showing no significant differences. Error bars represent the mean ± standard deviation of three independent experiments.

**Figure S10** Detection of cytoplasmic UDG levels in SK-N-AS, SK-N-BE (2), and 293T cells using a UDG ELISA kit. Error bars represent the mean ± standard deviation of three independent experiments. Abbreviations: BE (2), SK-N-BE (2); AS, SK-N-AS.

**Figure S11** Imaging-guided tumor resection after intratumoral injection of UUU-DZ-tFNA in SK-N-AS tumor-bearing mice.

**Figure S12** Serum levels of TBIL, AST, ALT, and ALP in mice detected before and 90 min after UUU-DZ-tFNA administration. Error bars denote SD from three independent parallel experiments; NS, no significant difference.

**Figure S13** (A) TEM image of exosome in plasma from NB patients. Scale bar: 100 nm. (B) Coomassie staining gel of proteins according to the exosomal EVS proteins. M, marker; L1, sample 1; L2, sample 2.

**Figure S14** (A) Detection of proteinase K protection assays using an ELISA kit. (B) Immunofluorescence co-localization analysis of EV UDG and the EV marker CD63. Merged channel demonstrates the overlapping distribution of UDG and exosomes. Scale bar = 200 nm. Abbreviation: EV, exosome.

**Figure S15** (A) UDG levels in plasma exosomes determined by UDG ELISA kit. (B) UDG levels in plasma exosomes determined by UUU-DZ-tFNA. (C) Correlation analysis between ELISA and UUU-DZ-tFNA measurements.

**Figure S16** Comparison of ROC curves for the CO-CPM model analyzed using 11 different machine learning algorithms. The x-axis represents the false positive rate (1 - specificity), and the y-axis represents the true positive rate (sensitivity). AUC (area under the curve) and its 95% confidence interval (CI) are indicated for each algorithm.

**Figure S17** Comparison of ROC curves for the CB-CPM model analyzed using 11 different machine learning algorithms. The x-axis represents the false positive rate (1 - specificity), and the y-axis represents the true positive rate (sensitivity). AUC (area under the curve) and its 95% confidence interval (CI) are indicated for each algorithm.

Table S1. DNA sequences used in the work

| **Name** | **DNA sequences (5' to 3')** |
| --- | --- |
| S1 | TTGACATCTGCAAGTGTTCTGTATTTTTTACATTCCTAAGTCTGAAACATTACAGCTTGCTACACGAGAAGAGCCGCCATAGTA |
| S2 | TCAGCCATCGAATAAACTAACTAATTTTTTATCACCAGGCAGTTGACAGTGTAGCAAGCTGTAATAGATGCGAGGGTCCAATAC |
| S3 | TCAGCCATCGAATAAACTAACTAATTTTTTCAACTGCCTGGTGATAAAACGACACTACGTGGGAATCTACTATGGCGGCTCTTC |
| S4 | TCAGCCATCGAATAAACTAACTAATTTTTTCAGACTTAGGAATGTGCTTCCCACGTAGTGTCGTTTGTATTGGACCCTCGCATC |
| S5 | ATACAGAACACTTGCAGATGTCAATTTCATCTCTTCTCCGAGCCGGTCGAAATAGTAAGCATCCCCG |
| S6 | CGGGGA/dU/GC/dU/dU/AC/dU/ATT/dU/CGACCGG |
| S7 | TTAGTTAGTTTATTCGATGGCTGA/BHQ3/CGCGAGCTTACTAT/rA/GGAAGAGATGTCGCG/Cy5/ |
| S8 | CGGGGATGCTTACTATTTCGACCGG |
| S9 | TTAGTTAGTTTATTCGATGGCTGA/BHQ3/CGCGAGCTTACTATAGGAAGAGATGTCGCG/Cy5/ |

Note: Fluorophore - quencher: Cy5 - BHQ3; dU, DeoxyUridine.

**Table S2 Comparison of the sensitivity, spatial resolution, clinical applicability, and LOD between reported fluorescence probes and UUU-DZ-tFNA**

| **Probe** | ***In vivo* detection** | **Rely on CHA/HCR** | **Spatial resolution** | **Target** | **LOD (mU/mL)** | **Ref** |
| --- | --- | --- | --- | --- | --- | --- |
| UAZ | Yes | No | Yes | UDG | 0.21 | [1] |
| Probe-dU | No | No | No | UDG | 250 | [2] |
| FNAs | No | No | Yes | UDG; APE1 | 1.2; 57 | [3] |
| CHA-DNAzyme  nanosystem | No | Yes | Yes | UDG | 0.23 | [4] |
| SEAD | No | No | No | UDG | 0.458 | [5] |
| HP | No | No | No | UDG | 0.368 | [6] |
| UUU-DZ-tFNA | Yes | No | Yes | UDG | 0.025 | This work |

**Reference**

[1]	Y. Jiang, Y. Chen, X. Gong, B. Weng, Y. He, F. Wang, *JACS Au* **2025**, *5* (9), 4288, <https://doi.org/10.1021/jacsau.5c00672>.

[2]	P. Zhang, F. He, X. Chang, C. Ren, *Acta Histochemica* **2025**, *127* (2), <https://doi.org/10.1016/j.acthis.2025.152252>.

[3]	X. Zhang, Y. Wu, X. Li, J. Liu, Y. Peng, L. Yuan, Y. Zhao, M. Liu, *Microchemical Journal* **2024**, *200*, <https://doi.org/10.1016/j.microc.2024.110383>.

[4]	X. Song, Q. Ding, J. Zhang, R. Sun, L. Yin, W. Wei, Y. Pu, S. Liu, *Analytical Chemistry* **2021**, *93* (40), 13687, <https://doi.org/10.1021/acs.analchem.1c03332>.

[5]	X. Q. Yuan, Y. M. Lei, Y. H. Li, X. M. Zhou, X. Yang, Y. Q. Chai, R. Yuan, Y. Zhuo, *Analytical Chemistry* **2024**, *96* (42), 17013, <https://doi.org/10.1021/acs.analchem.4c04477>.

[6]	C. Cui, G. Guo, T. H. Chen, *Biotechnology Journal* **2024**, *19* (7), e2400097, <https://doi.org/10.1002/biot.202400097>.

**Table S3.** Baseline characteristics of NB patients in the training cohort stratified by LIR-NB and HR-NB groups

| Variable | LIR (n = 19) | | HR (n = 11) | | t/Z | *P* |
| --- | --- | --- | --- | --- | --- | --- |
|  | n (%) | Median [IQR] | n (%) | Median [IQR] |  |  |
| Gender |  |  |  |  |  |  |
| Male | 12 (63) |  | 6 (54) |  |  | 0.643 |
| Female | 7 (37) |  | 5 (46) |  |  |  |
| *MYCN* |  |  |  |  |  |  |
| Amplified | 1 (5) |  | 5 (45) |  |  | 0.016 |
| No amplified | 18 (95) |  | 6 (55) |  |  |  |
| IDFRs |  |  |  |  |  |  |
| Positive | 12 (63) |  | 1 (9) |  |  | 0.007 |
| Negative | 7 (37) |  | 10 (91) |  |  |  |
| Age |  | 38.662 ± 29.806 |  | 48.064 ± 25.939 | -0.871 | 0.391 |
| NSE (ng/mL) |  | 30.300  [26.600 - 39.290] |  | 276.000  [52.800 - 753.000] | -2.776 | 0.005 |
| LDH (U/L) |  | 294.000  [260.000 - 344.000] |  | 929.300  [283.3000 - 1968.000] | -2.457 | 0.012 |
| UDG (U/mL) |  | 0.082 ± 0.012 |  | 0.095 ± 0.018 | -2.364 | 0.025 |

**Table S4.** Summary of performance data for the CO-CPM machine learning model

| **Model** | **Best_Sensitivity** | **Best_Specificity** | **Best_Cutoff** | **Best_Youden_Index** | **ROC** | **Accuracy** | **PPV** | **NPV** | **Kappa** | **F1** |
| --- | --- | --- | --- | --- | --- | --- | --- | --- | --- | --- |
| **glm** | 0.7272727 | 0.63157895 | 0.198989236 | 0.35885167 | 0.6531100 | 0.6666667 | 0.5333333 | 0.8000000 | 0.3333333 | 0.6153846 |
| **lda** | 0.9090909 | 0.57894737 | 0.187859919 | 0.48803828 | 0.7703349 | 0.7000000 | 0.5555556 | 0.9166667 | 0.4303797 | 0.6896552 |
| **glmnet** | 0.7272727 | 0.84210526 | 0.369288274 | 0.56937799 | 0.8229665 | 0.8000000 | 0.7272727 | 0.8421053 | 0.5693780 | 0.7272727 |
| **svmRadial** | 1.0000000 | 0.05263158 | 0.132748041 | 0.05263158 | 0.6220096 | 0.4000000 | 0.3793103 | 1.0000000 | 0.0391459 | 0.5500000 |
| **knn** | 0.8181818 | 0.63157895 | 0.214285714 | 0.44976077 | 0.7607656 | 0.7000000 | 0.5625000 | 0.8571429 | 0.4104803 | 0.6666667 |
| **rf** | 0.7272727 | 0.89473684 | 0.301000000 | 0.62200957 | 0.8277512 | 0.8333333 | 0.8000000 | 0.8500000 | 0.6341463 | 0.7619048 |
| **avNNet** | 0.9090909 | 0.63157895 | 0.205610086 | 0.54066986 | 0.8086124 | 0.7333333 | 0.5882353 | 0.9230769 | 0.4849785 | 0.7142857 |
| **nnet** | 0.7272727 | 0.94736842 | 0.571973869 | 0.67464115 | 0.8516746 | 0.8666667 | 0.8888889 | 0.8571429 | 0.7014925 | 0.8000000 |
| **naive_bayes** | 0.7272727 | 0.78947368 | 0.007907258 | 0.51674641 | 0.8181818 | 0.7666667 | 0.6666667 | 0.8333333 | 0.5070423 | 0.6956522 |
| **fda** | 0.8181818 | 0.26315789 | 0.132193735 | 0.08133971 | 0.6602871 | 0.4666667 | 0.3913043 | 0.7142857 | 0.0661479 | 0.5294118 |
| **rpart** | 0.8181818 | 0.68421053 | 0.133823529 | 0.50239234 | 0.7320574 | 0.7333333 | 0.6000000 | 0.8666667 | 0.4666667 | 0.6923077 |

Abbreviations: glm, Generalized Linear Model; lda, Linear Discriminant Analysis; glmnet, Generalized Linear Model via Regularization; svmRadial, Support Vector Machine with Radial Basis Function kernel; knn, K-Nearest Neighbors; rf, Random Forest; avNNet, Average Neural Network; nnet, Neural Network; naive_bayes, Naive Bayes Classifier; fda, Flexible Discriminant Analysis; rpart, Recursive Partitioning and Regression Trees.

**Uncropped gel images for Figure 2B in the main text.**

**Uncropped gel images for Figure 3B in the main text. The three sets of images shown herein represent technical replicates obtained from the identical experiment.**

**Uncropped gel images for Figure S2 in the Supporting Information.**

**Uncropped gel images for Figure S3 in the Supporting Information.**
